# Unlocking the potential of protein-derived peptides to target G-quadruplex DNA: from recognition to anticancer activity

**DOI:** 10.1093/nar/gkae471

**Published:** 2024-06-03

**Authors:** Francesco Merlino, Simona Marzano, Pasquale Zizza, Federica D’Aria, Nicola Grasso, Alice Carachino, Sara Iachettini, Annamaria Biroccio, Silvia Di Fonzo, Paolo Grieco, Antonio Randazzo, Jussara Amato, Bruno Pagano

**Affiliations:** Department of Pharmacy, University of Naples Federico II, Naples 80131, Italy; Department of Pharmacy, University of Naples Federico II, Naples 80131, Italy; Translational Oncology Research Unit, IRCCS-Regina Elena National Cancer Institute, Rome 00144, Italy; Department of Pharmacy, University of Naples Federico II, Naples 80131, Italy; Department of Pharmacy, University of Naples Federico II, Naples 80131, Italy; Translational Oncology Research Unit, IRCCS-Regina Elena National Cancer Institute, Rome 00144, Italy; Translational Oncology Research Unit, IRCCS-Regina Elena National Cancer Institute, Rome 00144, Italy; Translational Oncology Research Unit, IRCCS-Regina Elena National Cancer Institute, Rome 00144, Italy; Elettra-Sincrotrone Trieste S. C. p. A., Science Park, Trieste 34149, Italy; Department of Pharmacy, University of Naples Federico II, Naples 80131, Italy; Department of Pharmacy, University of Naples Federico II, Naples 80131, Italy; Department of Pharmacy, University of Naples Federico II, Naples 80131, Italy; Department of Pharmacy, University of Naples Federico II, Naples 80131, Italy

## Abstract

Noncanonical nucleic acid structures, particularly G-quadruplexes, have garnered significant attention as potential therapeutic targets in cancer treatment. Here, the recognition of G-quadruplex DNA by peptides derived from the Rap1 protein is explored, with the aim of developing novel peptide-based G-quadruplex ligands with enhanced selectivity and anticancer activity. Biophysical techniques were employed to assess the interaction of a peptide derived from the G-quadruplex-binding domain of the protein with various biologically relevant G-quadruplex structures. Through alanine scanning mutagenesis, key amino acids crucial for G-quadruplex recognition were identified, leading to the discovery of two peptides with improved G-quadruplex-binding properties. However, despite their *in vitro* efficacy, these peptides showed limited cell penetration and anticancer activity. To overcome this challenge, cell-penetrating peptide (CPP)-conjugated derivatives were designed, some of which exhibited significant cytotoxic effects on cancer cells. Interestingly, selected CPP-conjugated peptides exerted potent anticancer activity across various tumour types via a G-quadruplex-dependent mechanism. These findings underscore the potential of peptide-based G-quadruplex ligands in cancer therapy and pave the way for the development of novel therapeutic strategies targeting these DNA structures.

## Graphical Abstract

**Graphical Abstract:**
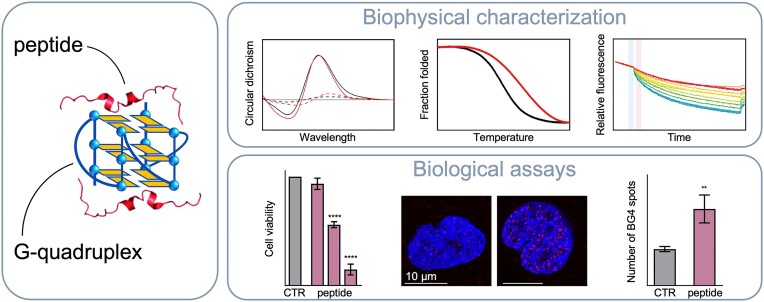

## Introduction

**Figure 1.**
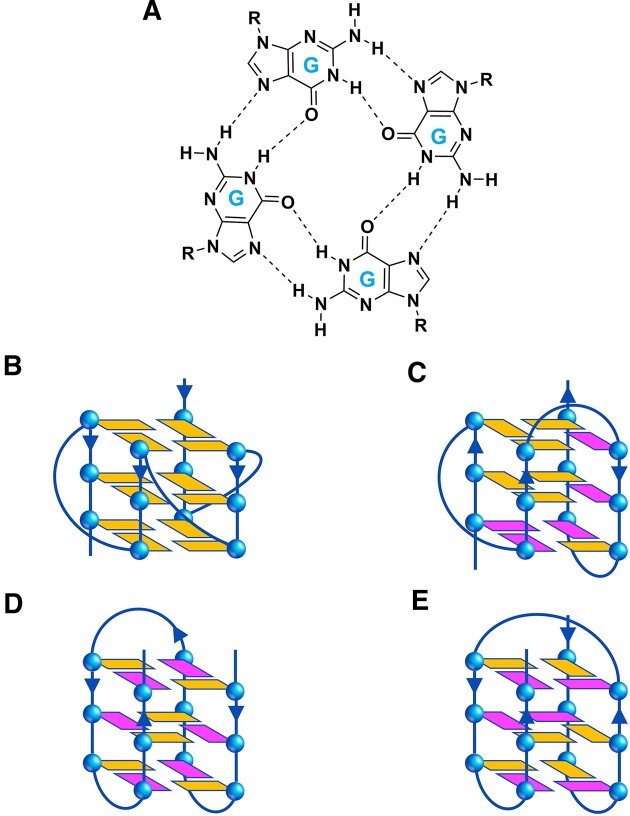
(**A**) Chemical structure of a G-tetrad showing the Hoogsteen hydrogen bonding between guanine bases, and schematic representation of the different G4 structural conformations investigated in this work: (**B**) parallel, (**C**) hybrid, and antiparallel (**D**) ‘chair’ and (**E**) ‘basket’ types. The *anti* and *syn* guanines are coloured in orange and magenta, respectively. The arrows indicate the direction of the DNA strands from the 5′ to 3′ end.

In addition to the double helix, DNA exhibits a now well-known propensity to adopt biologically relevant alternative secondary structures, including G-quadruplexes (G4s) (1). G4s represent a class of four-stranded nucleic acid structural arrangements resulting from the self-association of guanines (G) in G-rich sequences that form stacked G-tetrad structures (Figure 1). The *in vivo* existence of these noncanonical structures in human cells has been definitively confirmed through their visualization using structure-specific antibodies (2). A growing body of evidence indicates that these structures are involved in a variety of cancer-related processes, such as DNA repair, telomere maintenance and regulation of oncogene expression (1,3,4).

The primary challenge in anticancer drug research remains the identification of drugs with high selectivity and minimal side effects. Many conventional chemotherapeutic drugs exhibit significant toxicity on both normal and cancer cells, as they interact directly with the duplex DNA (5). The compelling evidence that G4 structures act, in concert with certain proteins (6–9), as active regulators of cancer-related genomic processes has made them an emerging research topic and an attractive target for gene regulation and anticancer drug design (1,3,10–13).

Crucial to this endeavour is the identification of molecules that specifically recognize G4s and modulate their stability to fine-tune their properties (11,13,14). Due to their peculiar architectures, such noncanonical DNA structures potentially offer a high degree of selective interactions, thus making them putative candidates for specific recognition processes. Indeed, they differ considerably from the B-DNA double helical structure in terms of the number and orientation of strands, groove width, and presence of loops. However, despite being excellent ligands *in vitro*, most G4-targeting bioactive small molecules reported so far showed unfavourable pharmacokinetics and toxicity due to poor selectivity, which critically hindered their advancement in chemotherapy (15,16). In addition, recent studies have questioned the effectiveness of certain small molecules in inhibiting the interactions between these noncanonical nucleic acids and proteins (17,18).

In this frame, the next generation of G4-targeting molecules should exhibit increased selectivity, enhanced ability to induce or resolve these noncanonical structures, and a greater degree of functionality. It would appear that traditional small molecule drugs are not able to fully guarantee these features. On the other hand, such intracellular DNA targets are difficult to target with protein-based therapeutics, which are likely capable of interacting with high specificity, but unable to cross cell membranes (19).

We reasoned that targeting these noncanonical structures using peptides may provide an exciting new avenue for the development of anticancer agents. Peptides can offer a unique combination of protein and small drug advantages. They can target the noncanonical DNA surface through interactions intrinsically complementary to the chemical features of the nucleic acids, ensuring high selectivity, as well as being potentially able to enter cells and reach intracellular targets with the same effectiveness as small molecules.

In this study, we explored whether it was possible to exploit the DNA recognition motif of a G4-binding protein to obtain peptides that selectively target G4s. To achieve this, we started from the crystal structure of a G4 in complex with the DNA-binding domain (DBD) of the yeast protein Rap1, reported by Traczyk *et al.* (20). Rap1 was among the earliest proteins to be discovered to also bind to and promote G4 formation *in vitro* (21). The Rap1-DBD consists of two homeodomain-like motifs, namely Myb1 and Myb2. In the crystal structure, only the Myb1 domain exhibits specific interactions with the G4, unlike Rap1’s binding mode to double-stranded DNA, where both Myb domains make essentially equivalent interactions with DNA. Therefore, aiming to develop G4-specific ligands, we investigated the interaction of a peptide (Myb^397–415^) derived from Myb1 domain with biologically relevant DNA G4s of different topologies [parallel, hybrid, or antiparallel (‘chair’ and ‘basket’ type)] (Figure 1) by using a combination of biophysical techniques.

We first evaluated the peptide's ability to bind and stabilize the G4 structures, then a complete thermodynamic characterization of the interaction between Myb^397–415^ and its most promising DNA targets was carried out. To further detail the interactions, point mutations were performed to identify the key amino acids in the G4-binding peptide. A library of nineteen Ala-monosubstituted derivatives, each of which differs from the parent peptide by a single amino acid replacement, was synthesized and investigated. Surprisingly, two of them exhibited even greater ability to bind and stabilize G4 DNA *in vitro* than Myb^397–415^.

However, the examination of the biological activity of the most promising peptides revealed their incapacity to significantly impact the viability of cancer cells, essentially due to their poor ability to penetrate the cell membrane. To overcome this issue, we designed and synthesized the Myb^397–415^ peptide conjugated to four different cell-penetrating peptides (CPPs). These are relatively short peptide sequences capable of crossing the cell membrane and carrying other molecules inside cells, addressing issues related to their intracellular targeting (22,23). Noteworthy, one of these CPPs demonstrated remarkable efficacy in facilitating cell entry and thereby enhancing the biological activity of Myb^397–415^. These findings have prompted a further investigation into the biological properties of this derivative, along with two other CPP-conjugated peptides selected from the Ala-scan library. Overall, the results presented here could help lay the groundwork for the development of innovative therapeutic strategies based on a new class of peptide-based G4 ligands.

## Materials and methods

### Materials

CPG supports, DNA phosphoramidites, and all reagents and solvents for oligonucleotide synthesis and purification were purchased from Merck KGaA (Darmstadt, Germany) and utilized without additional purification. MST capillaries were sourced from Nanotemper Technologies (München, Germany). All buffers were prepared using highly purified Milli-Q water and filtered before use. *N^α^*-Fmoc-protected amino acids, piperidine, trifluoroacetic acid (TFA), and 8-(9-fluorenylmethyloxycarbonyl-amino)-3,6-dioxaoctanoic acid (Fmoc-O2Oc) were purchased from IRIS Biotech GmbH (Marktredwitz, Germany). Activating and additive reagents, such as (1-cyano-2-ethoxy-2-oxoethylidenaminooxy)dimethylamino-morpholino-carbenium hexafluorophosphate (COMU) and ethyl cyano(hydroxyimino)acetate (Oxyma Pure), rink amide resin (0.63 mmol/g of loading substitution), *N,N*-diisopropylethylamine (DIEA), fluorescein isothiocyanate (FITC), and acetic anhydride were commercially obtained from Merck KGaA. Solvents for peptide synthesis, such as *N,N*-dimethylformamide (DMF), dichloromethane (DCM), diethyl ether (Et_2_O), and solvents for HPLC analyses and purifications, such as water, methanol (MeOH), and acetonitrile (ACN), were of reagent grade acquired from commercial sources (Merck KGaA or VWR International, Milan, Italy) and used without further purification.

### Oligonucleotide synthesis and sample preparation

DNA sequences (Table 1) were chemically synthesized on an ABI 394 DNA/RNA synthesizer (Applied Biosystem) at a 1-μmol scale using standard ß-cyanoethylphosphoramidite solid phase chemistry. DNA detachment from support and deprotection was achieved with a concentrated ammonia aqueous solution at 55°C for 17 h. Subsequently, the filtrates and washings were combined and concentrated under reduced pressure. Once redissolved in water, the DNA samples were purified using high-performance liquid chromatography (HPLC) with a Nucleogel SAX column (Macherey-Nagel, 1000-8/46). A 30-min linear elution gradient ranging from 100% buffer A to 100% buffer B was used at a flow rate of 1 ml/min. Buffer A consisted of an aqueous solution of 20 mM KH_2_PO_4_/K_2_HPO_4_ (pH 7.0), containing 20% (v/v) ACN, while buffer B was an aqueous solution of 1 M KCl, 20 mM KH_2_PO_4_/K_2_HPO_4_ (pH 7.0), containing 20% (v/v) ACN. Following purification, the oligonucleotides were desalted using C-18 cartridges (Sep-pak). The purity of the isolated oligonucleotides, confirmed by NMR, exceeded 98%. Then, the oligonucleotides were lyophilized and resuspended in 5 mM KH_2_PO_4_/K_2_HPO_4_ aqueous solution (pH 7.0), containing 20 mM KCl (or LiCl for *c-MYC*). Oligonucleotide concentrations were determined by UV absorption at 90°C using the molar extinction coefficient values ϵ (λ = 260 nm) derived from the nearest neighbor model (24). Finally, the DNA samples were subjected to an annealing procedure that involved heating at 90°C for 5 min, followed by gradual cooling to room temperature overnight and storage at 4°C for 24 h before use.

**Table 1.** List of DNA sequences used in this study, Myb^397–415^-induced thermal stabilization (Δ*T*_1/2_) for G4 and duplex DNA structures measured by CD melting experiments, and equilibrium dissociation constants (*K*_d_) for the binding of Myb^397–415^ to DNAs obtained by MST experiments

DNA	Sequence (5′ → 3′)	Structure	Δ*T*_1/2_(°C)^a^	*K*_d_(nM)
*c-KIT1*	AGGGAGGGCGCTGGGAGGAGGG	parallel G4	6.2 (±0.2)	340 (±44)
*c-KIT2*	CGGGCGGGCGCTAGGGAGGGT	parallel G4	11.2 (±0.2)	130 (±15)
*c-MYC*	TGAGGGTGGGTAGGGTGGGTAA	parallel G4	5.0 (±0.2)	430 (±100)
*HER2*	GGAGAAGGAGGAGGTGGAGGAGGAGGG	parallel G4	7.0 (±0.2)	130 (±20)
*HT-FANA*	TA**G**GGTTA**G**GGTTA**GG**GTTA**G**GG^b^	parallel G4	8.3 (±0.2)	140 (±26)
*HRAS1*	TCGGGTTGCGGGCGCAGGGCACGGGCG	antiparallel G4	–2.0 (±0.2)	n.d.^c^
*LWDLN1*	GGGTTTGGGTTTTGGGAGGG	antiparallel G4	–4.0 (±0.2)	n.d.^c^
*BCL-2*	GGGCGCGGGAGGAATTGGGCGGG	hybrid G4	8.0 (±0.2)	280 (±42)
*HT1*	TTGGGTTAGGGTTAGGGTTAGGGA	hybrid G4	1.6 (±0.2)	900 (±140)
*HT2*	TTAGGGTTAGGGTTAGGGTTAGGGTT	hybrid G4	2.7 (±0.2)	1180 (±90)
*VEGFR-17T*	GGGTACCCGGGTGAGGTGCGGGGT	hybrid G4	0.2 (±0.2)	n.d.^c^
*ds_12_*	CGCGAATTCGCG	duplex	1.0 (±0.2)	n.d.^c^

^a^Δ*T*_1/2_ represents the difference in melting temperature [Δ*T*_1/2_= *T*_1/2_ (DNA + peptide) – *T*_1/2_ (DNA)]. The *T*_1/2_ values of DNA alone are: *c-KIT1* = 53.8 (±0.1)°C, *c-KIT2* = 59.5 (±0.1)°C, *c-MYC*= 68.2 (±0.1)°C, *HER2* = 62.4 (±0.1)°C, *HT-FANA* = 62.9 (±0.1)°C, *HRAS1* = 52.3 (±0.1)°C, *LWDLN1* = 49.6 (±0.1)°C, *BCL-2* = 60.7 (±0.1)°C, *HT1* = 56.9 (±0.1)°C, *HT2* = 42.7 (±0.1)°C, *VEGFR-17T =* 53.7 (±0.1)°C, *ds_12_* = 62.7 (±0.1)°C. ^b^Bold G denotes 2′-fluoroarabino-guanosine (2′-F-ANA-guanosine). ^c^n.d. = not determined due to undetectable binding.

### Peptide synthesis

Peptides (Supplementary Table S1) were chemically produced by embracing the ultrasound-assisted solid-phase peptide synthesis (US-SPPS) combined with the 9-fluorenylmethoxycarbonyl (Fmoc)/*tert*-butyl (*t*Bu) orthogonal protection strategy (25). Detailed synthetic procedure is described in the Supplementary Data. The US-SPPS method was used for Fmoc-deprotection and coupling reactions, which were iteratively carried out until the resin-bound target peptide was obtained. The *N*-terminal primary amine of resin-bound sequences was acetylated, while, as regards the construction of fluorescein-labelled peptides used for MST experiments, it was treated with FITC after introducing of a spacer, such as O2Oc. Otherwise, cell-penetrating peptide sequences, such as Tat (RKKRRQRRR), YG-Tat (YGRKKRRQRRR), R_6_W_3_ (RRWWRRWRR) and R_7_W (RRRRRRRW), which were obtained by the same synthetic procedure described above and conjugated via a glycine–glycine spacer to the *N*-terminus of Myb^397–415^ and its selected derivatives, retain an unmodified primary amino function in that position. Following cleavage from the solid support, all crude peptides were purified via preparative reverse-phase high pressure liquid chromatography (RP-HPLC). Before biophysical and biological studies, each peptide was evaluated for purity (>95%) through HPLC analysis (Supplementary Figures S1-S29), as well as the correct molecular mass was confirmed through high-resolution mass spectrometry (HRMS) (LTQ Orbitrap) (Supplementary Table S1).

### Circular dichroism (CD) spectroscopy

CD experiments were carried out using a Jasco J-815 spectropolarimeter equipped with a PTC-423S/15 Peltier temperature controller. G4-forming and duplex-forming DNA molecules were prepared at a concentration of 2 μM in 5 mM KH_2_PO_4_/K_2_HPO_4_ buffer (pH 7.0) containing 20 mM KCl or LiCl. CD spectra of DNA/peptide mixtures were obtained by adding 1 mol equiv of peptide (stock solutions of 10 mM in H_2_O) with respect to the oligonucleotide. CD spectra of DNA molecules in the absence and presence of peptide were recorded at 20 and 100°C in the wavelength range of 220−320 nm and averaged over three scans, utilizing a scan rate of 100 nm/min with a 0.5 s response time and 1 nm bandwidth. The buffer baseline was subtracted from each spectrum. Additionally, CD spectra of Myb1^397–415^ in the absence and presence of G4s were recorded at 20°C in the 200−250 nm wavelength range using a scan rate of 20 nm/min, with a 4 s response time and 2 nm bandwidth. The G4 spectra were subtracted from the corresponding G4/peptide mixtures to obtain information on the peptide spectrum in the presence of G4. The percentage of secondary structures adopted by the peptide in the absence and presence of the G4s was estimated using the BeStSel software (26). CD melting experiments were performed in the absence and presence of peptides (1 or 2 mol equiv) in the 20−100°C temperature range at 1°C/min heating rate, monitoring changes of the CD signal at wavelengths corresponding to the maximum CD intensity for the G4s (264 nm for *c-KIT1*, *c-KIT2*, *c-MYC*, *HER2, HT-FANA, BCL-2*, and *VEGFR-17T*; 290 nm for *HRAS1*, *LWDLN1*, *HT1*, and *HT2*) and at 252 nm for *ds_12_* duplex. The apparent melting temperatures (*T*_1/2_) were determined using non-linear curve fitting with OriginLab© 2021 software (OriginLab Corp., MA, USA). Δ*T*_1/2_ values were calculated as the difference in the *T*_1/2_ values of the DNA structures in the presence and absence of a peptide. All experiments were performed in triplicate, and the reported values represent the average of three measurements.

### Microscale thermophoresis (MST)

MST experiments were conducted using a Monolith NT.115 instrument (NanoTemper Technologies, Munich, Germany) at 25°C with medium MST power settings. The FITC-labelled peptides were initially prepared at 1 μM in H_2_O and then diluted to 40 nM using the 5 mM KH_2_PO_4_/K_2_HPO_4_ buffer (pH 7.0) containing 20 mM KCl and supplemented with 0.1% Tween and 10% DMSO. DNA samples were also diluted using the same buffer supplemented with 0.1% Tween. During MST experiments, the concentration of the labelled peptide was kept constant at 20 nM, while a serial dilution of the investigated DNA molecules (ranging from 10 or 20 μM) was prepared and mixed with the peptide solution in a 1:1 volume ratio. All the samples, with a final concentration of 5% DMSO, were loaded into standard capillaries (NanoTemper Technologies) for MST measurements. Data analysis was carried out using the MO.Affinity Analysis (v2.3) software provided with the instrument.

### UV resonance Raman (UVRR) spectroscopy

UVRR measurements were conducted at the IUVS beamline of the Elettra Synchrotron Radiation facility (Trieste, Italy). An exhaustive description of the experimental apparatus is reported in the literature (27). UVRR experiments were acquired at 25°C with a 224 nm incident light from a table-top solid-state laser. Data were collected in back scattering geometry using a Czerny-Turner spectrometer (Trivista TR557, Princeton Instruments, USA) in the 700–4000 cm^−1^ range. The DNA concentration used for each experiment was 300 μM. The samples were placed in a quartz cuvette of 1 cm path length. The acquisition time was 8 h for each sample (G4, peptide and G4/peptide mixture). The solvent contribution was removed by normalization on the O–H stretching band of water, located above 3000 cm^−1^. To identify bands and assign contributions in the complex spectrum of DNA sequence, UVRR spectra of the single nucleotides (ATP, CTP, GTP and TTP) at 224 nm were also recorded. UV resonant bands were identified by comparison with literature data (28,29), including our previous works (30–32) and assigned to characteristic G4 molecular vibrations. Similarly, spectra of aromatic amino acids present in the peptide sequences were obtained. Spectra were reduced before data analysis.

### Isothermal titration calorimetry (ITC)

ITC experiments were conducted at 25°C using a nano-ITC Low Volume calorimeter (TA instruments, Lindon, UT, USA). DNA and peptide solutions were prepared using the same batch of buffer (5 mM KH_2_PO_4_/K_2_HPO_4_ buffer containing 20 mM KCl, pH 7.0) to prevent differences in buffer composition and pH. Each titration consisted of 25 injections of a 2 μl peptide solution (280–400 μM) sequentially injected using a computer-controlled 50 μl syringe, into the calorimetric vessel (190 μl) containing the oligonucleotide (16–20 μM). A spacing of 300 s between each injection was applied to allow the system to reach the equilibrium. The heat generated by peptide dilution was assessed in a control experiment by injecting the peptide solution into the buffer (Supplementary Figure S30). All measurements were performed in triplicate. Subsequently, the interaction heat for each injection was calculated considering the correction for the heat of peptide dilution. The corrected heat values were plotted as a function of the molar ratio to provide binding isotherms. The isotherms were fitted with a multiple-sites binding model using the NanoAnalyze software (TA instruments) provided with the instrument. This analysis provided the equilibrium binding constant (*K*_a_), binding enthalpy (Δ*H°*), and stoichiometry of interaction (*n*). The equilibrium dissociation constant (*K*_d_), Gibbs free-energy change and entropy change were determined using the following relationships: *K*_d_ = 1/*K*_a_, Δ*G°* = -*RT* ln *K*_a_ (*R* = 8.314 J mol^−1^ K^−1^, *T* = 298.15 K), and *T*Δ*S°* = Δ*H°* – Δ*G°*.

### Cells and culture condition

U2OS, HCT116 and MDA-MB-231 cells were purchased from the ATCC and grown in Dulbecco modified Eagle's medium (DMEM; EuroClone, Milan, Italy; ECM0728L) supplemented with 10% Fetal Bovine Serum (FBS, ThermoFisher Scientific/Gibco, Waltham, MA, USA; 10270-106), 2 mM l-glutamine and antibiotics in a humidified incubator at 37°C with 5% CO_2_ and atmospheric O_2_ concentration. Cell lines were authenticated and tested to be mycoplasma-free.

### Viability assay (crystal violet)

U2OS, HCT116, and MDA-MB-231 cells were seeded in 24-well plates at a density of 1 × 10^4^, 20 × 10^3^ and 12 × 10^3^ for each well, respectively. After 24 h, cells were treated with the indicated peptides and incubated at 37°C for additional 72 h. Upon treatment completion, cell medium was removed, the wells were washed twice in phosphate-buffered saline (PBS) and fixed with 4% formaldehyde for 15 min at room temperature (RT). After washing, 500 μl of crystal violet staining solution (Sigma-Aldrich, St. Louis, MO, USA) was added to each well and incubated for 30 min at RT. Finally, the plates were rinsed twice with water, air-dried and cell pellets were dissolved in 400 μl of a 10% aqueous solution of acetic acid. In total, 200 μl of each sample was transferred to a 96-well plate, and the optical density was measured at 570 nm (OD_570_) with an ELISA reader (Thermo Scientific, Waltham, MA, USA). The average absorbance in each condition was used to calculate the viability expressed as a percentage of treated *vs* untreated conditions (CTR).

### Immunofluorescence (IF) experiments

Cells were seeded on glass coverslips in a 24-well plate at a density of 1 × 10^5^ cells/well. After 24 h of treatment with the indicated peptides, cells were fixed in 4% formaldehyde in PBS for 10 min at RT, permeabilized with 0.5% Triton X-100 (5 min at RT), washed two times with PBS 1× and blocked with 3% BSA in PBS 1× for 2 h at RT. For immuno-labelling of G4 structures, cells were incubated with a mouse mAb that specifically recognizes DNA/RNA G4 structures (BG4 antibody 1:2000; Absolute Antibody, Oxford, UK, #Ab00174-1.1). After 2 h of incubation at RT, cells were washed three times with PBS 1× and then incubated for an additional 1 h with an antibody anti-mouse IgG (H + L), F(ab’)2 Fragment (Alexa Fluor 555 Conjugate 1:500; Cell Signaling Technology, Danvers, MA, USA, #4409S). Cells were then washed twice for 10 min with PBS 1× and nuclei were stained with Duolink® In Situ Mounting Medium with DAPI (Sigma-Aldrich, DUO82040). Fluorescence signals were recorded by using a Zeiss Laser Scanning Microscope 510 Meta (63× magnification) (Zeiss, Jena, Germany) and the number of G4 structures was quantified by using the software ImageJ (version 1.54f, National Institutes of Health, USA).

### Immuno-fluorescence *in situ* hybridization (Immuno-FISH)

To evaluate telomere-localized DNA damage, FISH assay with a telomeric probe was combined to IF staining of γH2AX. Cells were seeded on glass coverslips in a 6-well plate at a density of 1 × 10^5^ cells/well. After 24 h of treatment with indicated peptides, cells were washed three times with PBS 1×, fixed with formaldehyde 4% in PBS for 2 min at RT and washed three times with PBS 1×. The fixation protocol was repeated twice and then the samples were dehydrated by sequential exposure to 70%, 90% and 100% EtOH and DNA was denatured by incubation at 80°C for 3 min in the presence of the telomere probe solution (10 mM of Tris pH 7.2, MgCl_2_ buffer, 70% of deionized formamide, 0.5 μg/μl of TelC-Cy3 telomeric probe (Panagene, Daejeon, Korea), 0.25% of blocking reagent). Finally, the cells were incubated at RT in a humid chamber and, after 2 h, were washed for three times with the FISH solution (ddH_2_O, 0.1% BSA, Tris–HCl pH 7.2 and 50% formamide), permeabilized with 0.25% Triton X-100 in PBS 1×, washed two times with PBS 1× and blocked with 3% BSA in PBS 1× for 1 h at RT. Sequentially, cells were incubated with the mouse mAb anti-phospho-histone H2AX (γH2AX Ser139 1:300; Millipore, 05-636) in a humid chamber, overnight at 4°C. After two washes with 0.05% TritonX-100 in PBS 1×, cells were incubated with the secondary antibody anti-mouse IgG (H + L), F(ab’)2 Fragment (Alexa Fluor 488 Conjugate 1:500) for 1 h at RT. After two washes with PBS 1×, cells were sequentially dehydrated using 70%, 90%, and 100% EtOH, and upon drying, the nuclei were stained with Duolink® In Situ Mounting Medium with DAPI (Sigma-Aldrich, DUO82040). For quantitative analysis of γH2AX positive cells, at least 200 cells/condition were scored in triplicate. For Telomere-Induced Foci (TIFs) analysis, at least 25 γH2AX-positive cells on a single plane were scored. Cells with at least four telomere-γH2AX colocalization spots were considered TIF-positive (33). Fluorescence signals were recorded with Zeiss Laser Scanning Microscope 510 Meta (63× magnification) (Zeiss, Germany) and the experiment was analyzed with Adobe Photoshop CC 2019.

### RNA isolation and real-time RT‐PCR

U2OS cells were seeded on glass coverslips in a 6-well plate at a density of 2 × 10^5^ cells/well and treated with the peptides for 8, 16, and 24 h. Total RNA was isolated from the cell lines using WizPrep™ Total RNA Mini Kit (Cell). The quality and the quantity of RNA extraction was assessed by evaluating the *A*280/*A*260 nm and A260/A230 nm absorbance ratio (Nanodrop 1000, Thermo Fisher Scientific). The mRNA Reverse Transcription (RT) was performed using the QuantiTect Reverse Transcription Kit (Qiagen). mRNA expression was evaluated by SYBR Green (Applied Biosystems) in the QuantStudio 6 Flex Detection system (ThermoFisher Scientific). Primer sequences are indicated in Supplementary Table S2.

### Statistics

Biological experiments were replicated at least three times and the data were expressed as means ± standard deviation (SD). GraphPad Prism 8 software was used for the statistical analyses and the differences between groups were analysed by the unpaired Student's t-test. Differences were considered statistically significant for *P< 0.05; **P< 0.01; ***P< 0.001; ****P< 0.0001.

## Results and discussion

### Research design

Our study originated from the analysis of the crystal structure of the Rap1 DNA-binding domain (DBD) in complex with the ‘T-loops’ G4 (PDB ID: 6LDM) (20). This G4 structure adopts a parallel topology consisting of a stack of three G-tetrads, like most human G4s reported so far. The Rap1-DBD (residues 361–596) consists of two distinct homeodomain-like motifs (Myb1 and Myb2) connected by a linker. In the crystal structure, a specific interaction with the G4 is observed only for the Myb1 domain (20). Specifically, the recognition primarily occurs through the third α-helix of Myb1 (spanning residues Gly^400^ to Leu^411^), which is positioned diagonally across an external G-tetrad of the G4, almost entirely covering the exposed hydrophobic surface of the guanine bases. In addition to the hydrophobic/stacking and polar interactions involving amino acid sidechains, the complex is further stabilized by a network of direct and water-mediated hydrogen bonds. The Thr^399^ residue located just outside the α-helix also participates by making contacts with the ribose-phosphate backbone of the G4.

Therefore, in this study we first focused on a 19 amino acid-long peptide sequence encompassing the G4-binding region of Myb1, specifically ranging from residues 397 to 415 (NHTGNSIRHRFRVYLSKRL, expected net charge 5.18 at pH 7.0), referred to as Myb^397–415^. The secondary structure of Myb^397–415^ in solution was evaluated by circular dichroism (CD) spectroscopy at 25°C. The CD spectrum of the peptide displayed very low ellipticity above 210 nm and a negative band near 200 nm (Supplementary Figure S31), thus indicating that it predominantly adopts a random conformation under the experimental conditions employed.

To investigate the G4-binding properties of Myb^397–415^, a panel of human G4-forming sequences was selected (Table 1). Since Rap1 is a highly evolutionarily conserved protein found at telomeres and plays an important role in telomere end protection (34,35), we specifically selected various G4-forming human telomeric sequences capable of adopting different G4 topologies. Indeed, the *in vivo* conformation(s) of the human telomeric G4 remains elusive and undetermined. Given the molecularly crowded intracellular environment (36) and its specific recognition by telomerase (17), the parallel topology emerges as the most likely candidate for playing a relevant biological role among all telomeric G4 conformations (37–39). However, it is also possible that different conformations exist at different telomeric overhangs within a cell (40). Accordingly, we investigated two G4-forming human telomeric sequences, *HT1* and *HT2*, with *HT1* assuming a pure hybrid-1 conformation and *HT2* primarily adopting a hybrid-2 conformation (Supplementary Figure S32) (41). Furthermore, we also employed a telomeric sequence (*HT-FANA*) incorporating the *anti*-favouring 2′-fluoroarabino-guanosine (2′-F-ANA-guanosine) in the positions that adopt *syn* conformation in the hybrid-1 form enabling the conformational switch from the hybrid to the parallel folding topology (42).

To expand our investigation and evaluate the ability of the peptide to discriminate between various G4 structures, we also included a series of G4-forming sequences identified in the promoter regions of the *BCL-2*, *c-KIT (c-KIT1*, and *c-KIT2)*, *c-MYC*, *HER2*, *HRAS* (*HRAS1*) and *VEGFR-2* (*VEGFR-17T*) genes (4,43–49), and the *LWDLN1* sequence (50). These sequences adopt diverse G4 topologies, namely parallel, antiparallel, and hybrid (see Table 1 for details), and some of them have quite unique folding characteristics (Supplementary Figure S32). In addition, a 12-nucleotide self-complementary duplex-forming sequence (*ds_12_*) was included to assess the selectivity of the peptide for G4 over duplex DNA.

### Investigating Myb^397–415^ interaction with G4 structures via CD, MST, and UVRR experiments

CD spectroscopy is a primary tool for characterizing G4 topologies in solution (51). Indeed, G4 structures with different polarities exhibit different CD spectral features, particularly in the range of 220–320 nm, which is diagnostic of the G4 motifs. CD is also useful for quickly assessing ligand binding to a G4 structure and evaluating its effects on the native folding topology of the G4 (52). This is achieved by directly comparing the spectra of the G4 with and without the ligand. Therefore, CD spectra of each G4 were first recorded in the absence of Myb^397–415^ (Supplementary Figure S33). The results showed that *c-KIT1*, *c-KIT2*, *c-MYC*, *HER2* and *HT-FANA* all adopted a parallel G4 conformation. This was evidenced by the presence in their CD spectra of a positive band at around 264 nm and a negative one at 240 nm. On the other hand, *HRAS1* and *LWDLN1* sequences exhibited the typical CD profile of antiparallel G4 structures, characterized by a positive band at around 295 nm and a negative one at 260 nm. Finally, the CD spectra of *HT1*, *HT2*, *BCL-2* and *VEGFR-17T* exhibited the characteristic signatures of hybrid and mixed parallel/antiparallel G4 conformations, with two positive bands at around 290 and 265 nm, and a negative one at around 240 nm. Next, DNA/peptide mixtures were prepared by adding Myb^397–415^ to the G4 structures in solution and CD spectra were recorded. The results revealed that Myb^397–415^ had minimal or negligible effect on the CD spectra of the investigated G4-forming sequences (Supplementary Figure S33), indicating no structural rearrangement of the G4s in the presence of the peptide.

To ascertain the interaction between Myb^397–415^ and the G4s and gain quantitative data on binding affinity, we conducted MST experiments. MST allows for the examination of the thermophoretic behaviour of a fluorescent molecule (intrinsically fluorescent or labelled with a fluorescent probe) in small temperature gradients. This behaviour is significantly affected by alterations in size, charge, and/or hydration shell resulting from the binding of any interacting partners (53). As a result, changes in the thermophoretic behaviour of the target molecule can be used to achieve the equilibrium dissociation constant (*K*_d_). For such experiments, serial dilutions of DNA molecules were prepared, mixed with a constant concentration of *ad hoc* synthesized FITC-labelled Myb^397–415^, loaded into capillaries, and subsequently analysed. The resulting binding curves provided clear evidence of peptide binding to almost all G4 structures and revealed particularly strong affinity for certain G4s (Table 1 and Supplementary Figure S34). Indeed, Myb^397–415^ showed the highest affinity for *c-KIT2*, *HER2*, and *HT-FANA* G4s (*K*_d_ = 130, 130, and 140 nM, respectively), all adopting parallel G4 conformations. Furthermore, the peptide exhibited good affinity for the hybrid-type *BCL-2* and for the parallel-stranded *c-KIT1* and *c-MYC* G4s (*K*_d_ = 280, 340, and 430 nM, respectively), while it showed a lower affinity for the hybrid-type telomeric G4s represented by *HT1* and *HT2* (*K*_d_ = 900 and 1180 nM, respectively). Conversely, Myb^397–415^ showed only negligible binding to the antiparallel G4 structures formed by the *HRAS1* and *LWDLN1* sequences, as well as to the mixed parallel/antiparallel G4 structure adopted by *VEGFR-17T*.

To assess if these interactions lead to the stabilization of DNA structures, we performed CD melting experiments to measure the peptide-induced changes in the melting temperature (Δ*T*_1/2_) of the various G4s (Supplementary Figure S35). Indeed, if a peptide (or any other ligand) bound to a G4 dissociates from the latter before its unfolding, it would not be able to thermally stabilize the DNA structure. Therefore, if stabilization of a G4 structure is observed, it can be inferred that ligand dissociation is concomitant with the unfolding of the DNA structure. Conversely, if a peptide has higher affinity to the single-stranded DNA than to G4 structure, it should result in destabilization of the G4. The results of these experiments, summarized in Table 1, reveal that Myb^397–415^ (1 mol equiv) induces thermal stabilization in most G4s, with the exception of *HRAS1*, *LWDLN1* and *VEGFR-17T*. The most significant thermal stabilization effects were observed for *c-KIT2* and *HT-FANA* (Δ*T*_1/2_ = 11.2 and 8.3°C, respectively), followed by *BCL-2*, *HER2*, *c-KIT1* and *c-MYC*. On the other hand, the peptide exhibited only a very weak stabilization of hybrid-type telomeric G4s (Δ*T*_1/2_ ≤ 2.7°C). Overall, these findings align with those from MST experiments and suggest that Myb^397–415^ has some preference for parallel G4 motifs.

**Figure 2.**
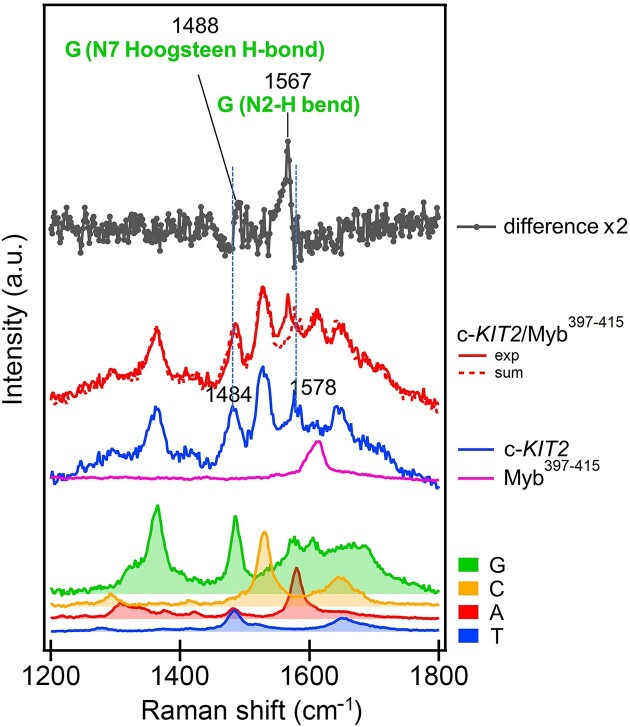
UVRR spectra to gain insights into the binding mode of Myb^397–415^ to *c-KIT2* G4. From bottom to top: thymine (blue), adenine (red), cytosine (orange) and guanine (green) (as constituents of a nucleotide) normalized and weighted according to the respective number of nucleotides present in the *c-KIT2* sequence; Myb^397–415^ (magenta); *c-KIT2* (blue); *c-KIT2*/Myb^397–415^ complex (red); arithmetic sum of *c-KIT2* and Myb^397–415^ spectra (red-dashed); normalized difference between the spectra of the complex and the arithmetic sum (dark grey). Spectra were normalized to the intensity of the *c-KIT2* spectrum.

UV resonance Raman (UVRR) spectroscopy was then employed to get some insights into the binding mode of Myb^397–415^ to G4-forming DNA (*c-KIT2* was used as G4 model). An in-depth analysis was conducted on the peaks assigned to specific groups of nucleobases and aromatic amino acids, focusing in particular on their changes upon binding. UVRR spectra of the G4, peptide, and corresponding complex collected at *T* = 25°C are shown in Figure 2 along with the difference between the spectrum of the complex and that corresponding to the arithmetic sum of constituents to emphasize the spectral perturbations induced by the interaction. Alterations in band intensity and/or position indicate that an interaction is happening and suggest the structural moieties involved. UVRR spectral perturbations provided evidence that the peptide binds to the surface of external G-tetrads. Indeed, the presence, in the difference spectrum, of a blue shift of the guanine N7 Hoogsteen H-bond band, together with the red shift and increase in intensity of the band associated with the guanine N2-H bending, indicate that such guanine residues take part in the interaction.

Furthermore, to evaluate the selectivity of Myb^397–415^ for G4 structures over double-stranded DNA, CD experiments were performed using the 12-nucleotide self-complementary *ds_12_* sequence as a representative duplex model. The CD spectrum of *ds_12_* typically displays a positive band at around 280 nm and a negative one at around 250 nm (Supplementary Figure S33). These bands showed no significant alteration upon peptide addition, indicating no unfolding or structural rearrangement of the DNA molecule due to the peptide. Moreover, the thermal stability of the duplex was essentially unaffected by the peptide (Δ*T*_1/2_ = 1.0°C), showing a preference of Myb^397–415^ for G4 structures over duplex (Supplementary Figure S35 and Table 1). This observation is corroborated by MST data, which revealed negligible Myb^397–415^ binding to *ds_12_* duplex, providing further evidence of its selectivity for G4 over duplex DNA.

### Thermodynamic data for the binding of Myb^397–415^ to G4s by ITC analysis

To conduct an in-depth investigation into the interaction between Myb^397–415^ and selected G4 structures from a thermodynamic point of view, an ITC analysis was performed (54). Recognized as one of the most reliable methods for characterizing the thermodynamics of binding interactions of biological macromolecules, ITC stands out as the only technique capable of directly quantifying both the enthalpic and entropic aspects of an interaction without making any assumptions (55,56). This capability allows for a detailed elucidation of the thermodynamic driving forces governing molecular recognition. For this study, *c-KIT2* and *HER2*, two of the parallel G4s for which Myb^397–415^ has demonstrated high affinity, were selected as representative models.

**Figure 3.**
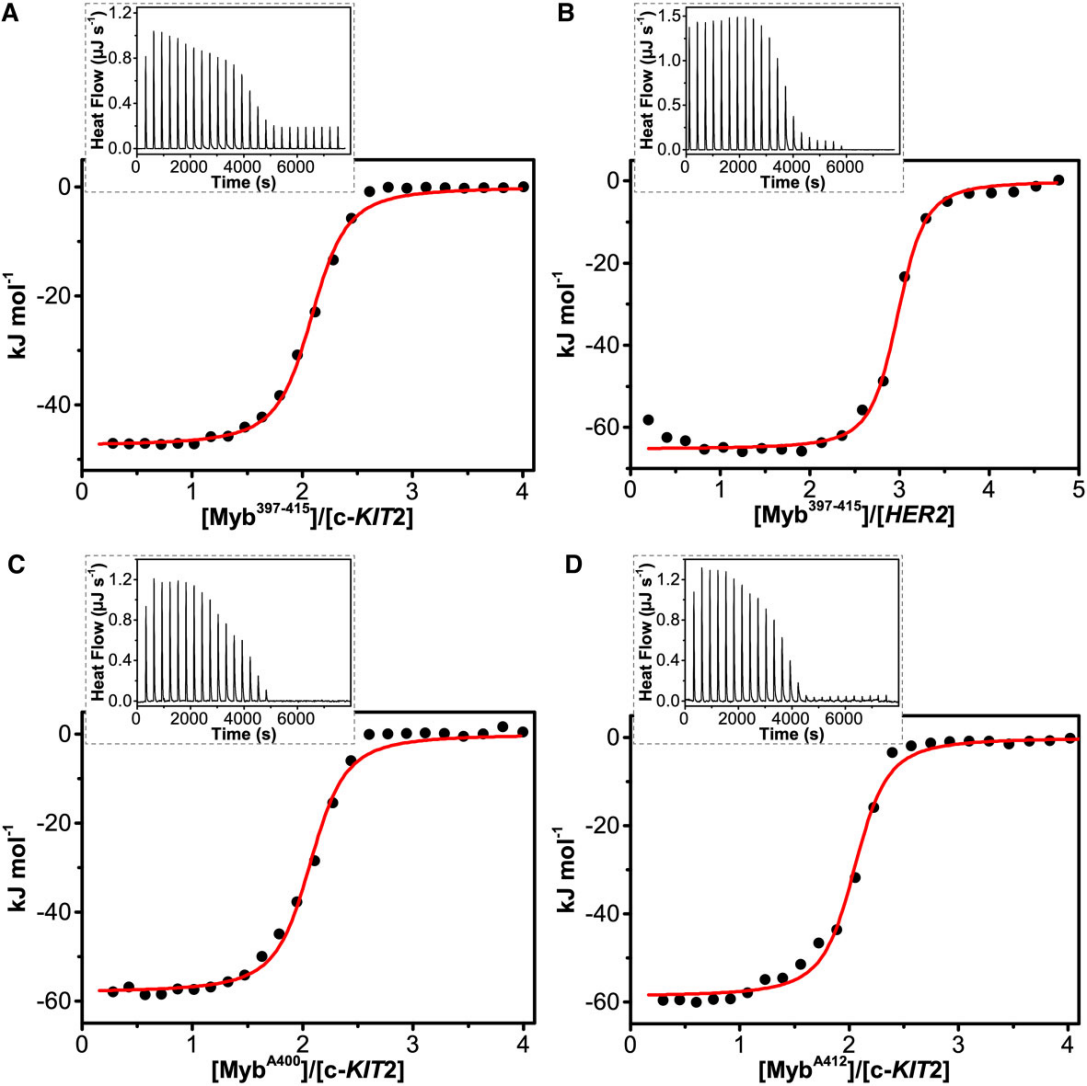
Raw ITC data (insets) and binding isotherms for titration of (**A**) *c-KIT2* and (**B**) *HER2* G4s with Myb^397–415^ peptide, and of *c-KIT2* with (**C**) Myb^A400^ and (**D**) Myb^A412^ obtained at 25°C. The black dots represent the experimental data obtained by integrating the raw ITC data and subtracting the heat of peptide dilution into the buffer. The red lines represent the best-fit curve for the binding.

Figure 3 shows the raw ITC data (insets) and binding isotherms for the interaction of the peptide with the two G4s, clearly indicating exothermic binding processes. To determine the heat of peptide dilution, a control experiment was also carried out by injecting the peptide solution into the buffer (Supplementary Figure S30). Thermodynamic analysis revealed an enthalpically driven interaction of the peptide with both G4s (Δ*H°* < 0), accompanied by an unfavourable entropic contribution (*T*Δ*S°* < 0) (Table 2). The magnitude of these thermodynamic contributions differs among the G4s, indicating some differences in complex formation. The estimated equilibrium binding constants (*K*_a_) were 5.0 × 10^6^ and 1.0 × 10^7^ M^−1^ for *c-KIT2* and *HER2* G4s, respectively. The thermodynamic signatures suggest that the driving force behind the binding process is the formation of new interactions between the peptide and DNA molecules. On the other hand, opposing entropic contributions imply that resulting complexes exhibit increased rigidity compared to free molecules. Given the absence of observable structural changes in the G4s, this may suggest that upon binding to DNA, the peptide may undergo some structural change from its completely random conformation when free in solution.

**Table 2.** Thermodynamic parameters for the peptide-G4 interactions obtained by ITC at 25°C

	Myb^397–415^		Myb^A400^	Myb^A412^
	*c-KIT2*	*HER2*	*c-KIT2*	*c-KIT2*
***K*_a_ (M^−1^)**	5.0 (±0.3) ×10^6^	1.0 (±0.1) ×10^7^	7.3 (±1.3) ×10^6^	5.3 (±0.8) ×10^6^
***K*_d_ (nM)**	200 (±12)	100 (±10)	137 (±24)	187 (±28)
Δ***G*° (kJ mol^−1^)**	–38.2 (±2.3)	–40.0 (±4.0)	–39.2 (±7.0)	–38.4 (±5.8)
Δ***H*° (kJ mol^−1^)**	–47.4 (±0.8)	–64.4 (±1.1)	–60.2 (±2.0)	–58.8 (±1.4)
***T*** Δ***S*° (kJ mol^−1^)**	–9.2 (±3.1)	–24.4 (±5.1)	–21.0 (±9.0)	–20.4 (±7.2)
***n***	2	3	2	2

To investigate potential conformational changes in Myb^397–415^ upon binding to G4s, we analysed the CD spectra of the peptide in the presence of the DNA molecules within the wavelength range of 200–250 nm. By subtracting the CD signal of the G4s from the spectrum of the corresponding G4/peptide mixture, we evaluated any conformational changes in Myb^397–415^ upon interaction with G4s (Supplementary Figure S36). Interestingly, the CD spectrum revealed the appearance of an α-helical signal in the presence of both G4s, indicating an increase in the estimated helical content by approximately 10 and 20% in the case of *c-KIT2* and *HER2*, respectively.

### Alanine scanning analysis identified two peptides more effective than Myb^397–415^

To further analyse the interactions and identify the key residues involved in the binding of Myb^397–415^ to G4-forming DNA, a mutagenesis analysis was conducted. This approach involved the systematic replacement of each residue of the Myb^397–415^ sequence, one at a time, with the non-bulky and chemically inert alanine residue. For this purpose, nineteen single Ala-substituted peptides were synthesized (Table 3), and the impact of each specific residue on the stabilization of *c-KIT2* G4 was assessed by CD melting assay (Supplementary Figure S37). Experiments were performed using 2 mol equiv of each Ala-substituted peptide with respect to G4.

**Table 3.** List of Ala-substituted peptide sequences compared to Myb^397–415^ and relative peptide-induced thermal stabilization (Δ*T*_1/2_) of *c-KIT2* G4, assessed by CD melting experiments performed using 2 mol equiv of each peptide with respect to G4

Name	Sequence (N_term_ → C_term_)	Δ*T*_1/2_ (°C)^a^
Myb^397–415^	NHTGNSIRHRFRVYLSKRL	17.3 (±0.2)
Myb^A397^	**A**HTGNSIRHRFRVYLSKRL	18.0 (±0.2)
Myb^A398^	N**A**TGNSIRHRFRVYLSKRL	19.8 (±0.2)
Myb^A399^	NH**A**GNSIRHRFRVYLSKRL	18.5 (±0.2)
Myb^A400^	NHT**A**NSIRHRFRVYLSKRL	21.1 (±0.4)
Myb^A401^	NHTG**A**SIRHRFRVYLSKRL	19.7 (±0.2)
Myb^A402^	NHTGN**A**IRHRFRVYLSKRL	18.5 (±0.2)
Myb^A403^	NHTGNS**A**RHRFRVYLSKRL	17.7 (±0.2)
Myb^A404^	NHTGNSI**A**HRFRVYLSKRL	16.6 (±0.2)
Myb^A405^	NHTGNSIR**A**RFRVYLSKRL	16.0 (±0.2)
Myb^A406^	NHTGNSIRH**A**FRVYLSKRL	12.1 (±0.3)
Myb^A407^	NHTGNSIRHR**A**RVYLSKRL	17.8 (±0.2)
Myb^A408^	NHTGNSIRHRF**A**VYLSKRL	14.0 (±0.6)
Myb^A409^	NHTGNSIRHRFR**A**YLSKRL	14.7 (±0.6)
Myb^A410^	NHTGNSIRHRFRV**A**LSKRL	13.6 (±0.2)
Myb^A411^	NHTGNSIRHRFRVY**A**SKRL	19.5 (±0.4)
Myb^A412^	NHTGNSIRHRFRVYL**A**KRL	23.3 (±0.2)
Myb^A413^	NHTGNSIRHRFRVYLS**A**RL	10.7 (±0.6)
Myb^A414^	NHTGNSIRHRFRVYLSK**A**L	10.7 (±0.6)
Myb^A415^	NHTGNSIRHRFRVYLSKR**A**	17.8 (±0.5)

^a^Δ*T*_1/2_ represents the difference in melting temperature [Δ*T*_1/2_= *T*_1/2_ (DNA + peptide) – *T*_1/2_ (DNA)]. *T*_1/2_ of *c-KIT2* alone = 59.5 (±0.1)°C.

**Figure 4.**
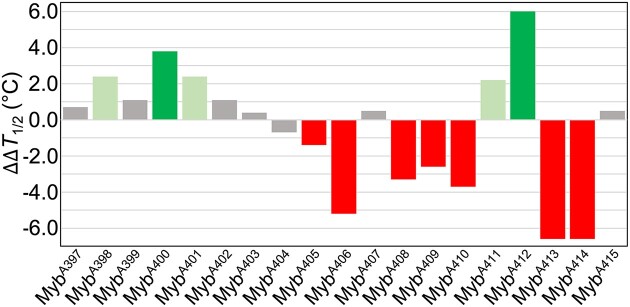
Bar graph depicting changes in *c-KIT2* G4-stabilizing properties (ΔΔ*T*_1/2_) of the indicated Ala-modified peptides with respect to Myb^397–415^, as determined by CD melting assay. The error in ΔΔ*T*_1/2_ values does not exceed 0.8°C.

The results of this analysis revealed that replacement of H405, R406, R408, V409, Y410, K413 and R414 with an alanine residue resulted in a significant decrease in the G4-stabilizing properties of the corresponding Ala-replaced peptide (Figure 4), suggesting their strong involvement in the binding to the G4. On the other hand, negligible differences were observed when residues N397, T399, S402, I403, R404, F407 and L415 were mutated, thus indicating that their substitution does not have a significant impact on the interaction with the G4 structure. Replacement of H398, N401 and L411 with alanine resulted in a small increase in the ability of the peptide to stabilize the G4 (ΔΔ*T*_1/2_ ≤ 2.4°C). Surprisingly, substitution of G400 and S412 produced a significant increase in the G4-stabilizing properties of the corresponding Ala-modified peptides (referred to as Myb^A400^ and Myb^A412^) with an increase, compared to Myb^397–415^, of 3.8 and 6.0°C for Myb^A400^ and Myb^A412^, respectively (Figure 4).

To confirm the binding of these two peptides to the G4 by direct measurements and characterize the energetics of the interactions, MST and ITC experiments were performed. The results of MST measurements showed that Myb^A400^ and Myb^A412^ peptides (labelled with FITC for these experiments) are both able to bind to *c-KIT2* G4, with *K*_d_ values of around 170 (±25) and 130 (±20) nM, respectively (Supplementary Figure S38). ITC measurements confirmed that Myb^A400^ and Myb^A412^ interact with *c-KIT2* G4 *in vitro* (Figure 3) with high affinity (*K*_d_ = 137 and 187 nM, respectively) (Table 2). In addition, the data indicate that Myb^A412^ has an affinity to the G4 comparable to that of Myb^397–415^, while Myb^A400^ shows a slightly higher affinity than Myb^397–415^. Interestingly, similar thermodynamic signatures are observed for these interactions, suggesting analogous binding modes. However, the magnitude of enthalpic and entropic contributions suggests the formation of some additional interactions between these peptides and the DNA, as indicated by the more favourable enthalpy changes for Myb^A400^ and Myb^A412^ peptides (–60.2 and –58.8 kJ mol^−1^, respectively) compared to Myb^397–415^ (–47.4 kJ mol^−1^).

### Investigation of the anticancer properties of Myb^397–415^ and its derivatives

**Figure 5.**
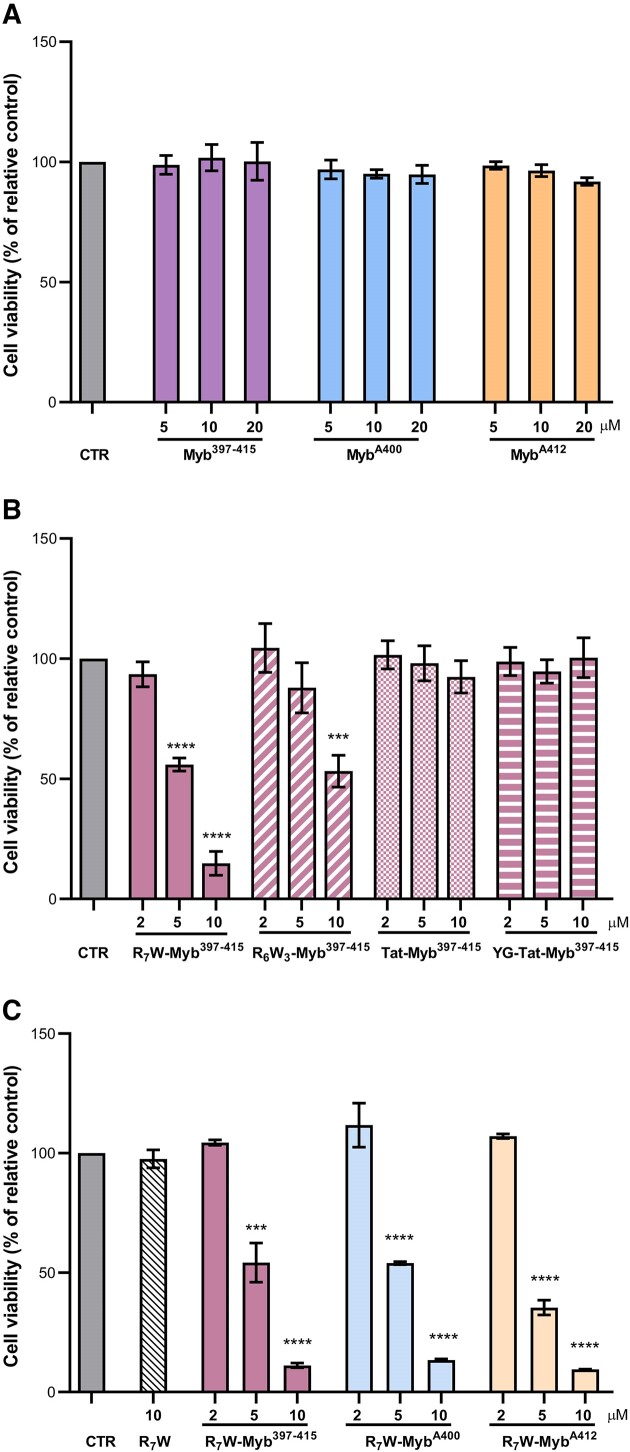
Selected peptides impair tumour cell viability. ALT-positive U2OS osteosarcoma cells were treated for 72 h with the indicated concentrations of the selected peptides, and cell viability was determined by crystal violet assay. (**A**) The Myb^397–415^ peptide and its derivatives (Myb^A400^ and Myb^A412^) were tested in their native form; (**B**) Myb^397–415^ was conjugated to four different CPPs (R_6_W_3_, R_7_W, Tat and YG-Tat) and the resulting molecules were evaluated; (**C**) Peptides were conjugated with R_7_W and the resulting derivatives (R_7_W-Myb^397–415^, R_7_W-Myb^A400^ and R_7_W-Myb^A412^) were tested. Results are expressed as the percentage of viable cells in treated samples over their untreated counterpart (CTR). Histograms show mean values ± SD of three independent experiments performed in triplicate; ***P< 0.001, ****P< 0.0001.

Based on the results of biophysical analyses, we pointed at investigating the antitumoral potential of the Myb^397–415^ peptide and its most promising derivatives (Myb^A400^ and Myb^A412^). Surprisingly, crystal violet experiments conducted in U2OS, a well-established osteosarcoma cell model, revealed that the investigated peptides were unable to significantly impact cancer cells viability (Figure 5A). Since the effectiveness of peptide-based therapeutics often depends on their ability to efficiently penetrate cells (57,58), we designed and synthesized chemically-modified derivatives of the parent Myb^397–415^ peptide. In these derivatives, Myb^397–415^ was *N*-terminally conjugated to different additional peptide fragments, namely R_6_W_3_, R_7_W, Tat and YG-Tat (see Materials and methods section for details) which were selected as well-known and/or U2OS-effective cell-penetrating peptides (CPPs) (23,59,60). We envisioned that bioconjugation to CPPs could provide a unique strategy to enhance the permeability of these molecules. Indeed, CPPs are short peptides that enter cells and are able to carry other molecules inside cells (58,61), thus overcoming one of the main rate-limiting steps in the development of many therapeutic substances, as demonstrated by studies showcasing their efficacy in delivering therapeutic molecules.

We evaluated the capability of such CPP-conjugated peptides to impair tumour cell growth. Interestingly, the two derivatives of Myb^397–415^ obtained by conjugating the peptide with R_7_W and R_6_W_3_ were found to impair tumour viability with an IC_50_ (the value corresponding to the concentration of drug capable of killing 50% of cells) of about 5 and 10 μM, respectively (Figure 5B).

Since conjugation with R_7_W resulted particularly effective in promoting the biological activity of Myb^397–415^, we pointed at evaluating if the introduction of this specific carrier would affect the G4-stabilizing properties of the peptide. Interestingly, CD experiments evidenced no significant differences between Myb^397–415^ and its modified form (R_7_W-Myb^397–415^) (Supplementary Figure S39), suggesting that the conjugation just promotes the entry of the molecule within the cells. Based on these results, all the peptides were conjugated with the selected carrier, R_7_W, and the obtained molecules were subjected, once again, to biological screening. As reported in the Figure 5C, R_7_W-Myb^A400^ and R_7_W-Myb^A412^ inhibited tumour cell growth essentially to the same extent as R_7_W-Myb^397–415^, with R_7_W-Myb^A412^ appearing to be slightly more effective than the others at the 5 μM concentration.

Of note, U2OS belong to a subcategory of tumour cells (accounting for about the 15% of total cancers), characterized by the capability to maintain telomeres length independently from telomerase activity (62,63). As reported in the literature (64,65), these telomerase-negative models—collectively referred to as ALT (alternative lengthening of telomeres) cells—are more sensitive than telomerase-positive cells to treatment with ligands capable of binding and stabilizing G4s (66,67). Based on these observations, the antitumoral activity of the synthetized R_7_W-conjugated peptides was also evaluated in telomerase-positive cells. As evidenced from these additional experiments, peptides showing effectiveness in the U2OS model resulted, at slightly higher concentrations (IC_50_ ≈ 10 μM), able to impair the viability of both HCT116 and MDA-MB-231 (Supplementary Figure S40), two telomerase-positive cell models deriving from colorectal and breast cancer, respectively. Additionally, being the evaluated cell lines all proficient for BRCA1 and BRCA2, two genes with a key role in homologous recombination and DNA repair (68), it is also possible to assert that these peptides, unlike most known G4 ligands which are mainly effective in BRCA-deficient models (1,69,70), promote their activity also in tumour cells able to potentially repair DNA damage. In summary, this first set of biological data clearly indicates that R_7_W-Myb^397–415^, R_7_W-Myb^A400^ and R_7_W-Myb^A412^ are able to exert a potent antitumoral activity in an array of tumour histotypes, independently from the molecular mechanisms of telomere maintenance and DNA repair.

**Figure 6.**
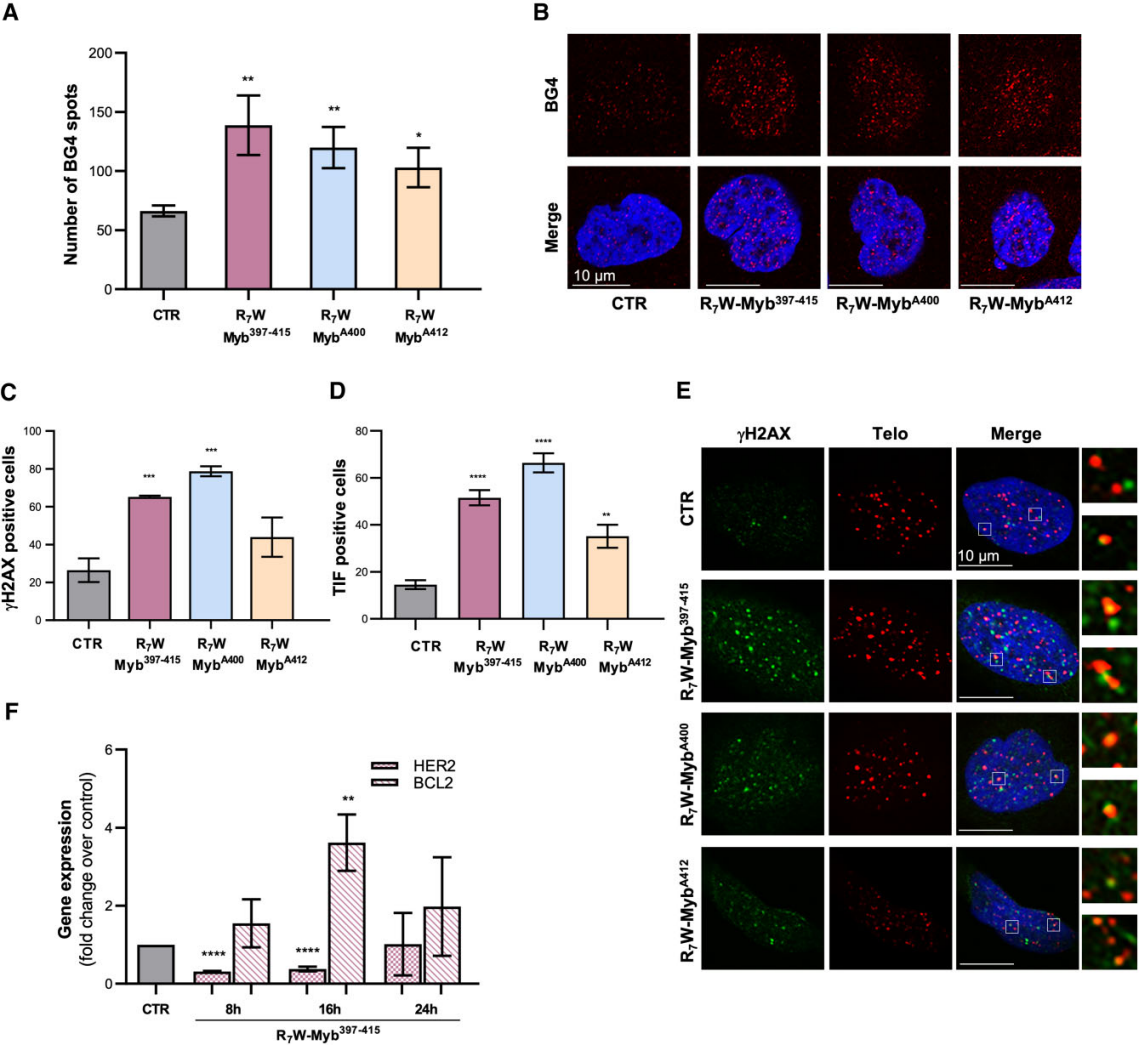
Mechanism of action of the selected peptides. (A, B) U2OS cells were subjected to 24 h of treatment with 10 μM of R_7_W-Myb^397–415^, R_7_W-Myb^A400^ and R_7_W-Myb^A412^ and G4-stabilizing activity was evaluated by IF confocal microscopy through the use of an antibody (BG4) able to specifically recognize G4 structures in cells. (**A**) Histograms showing the number of BG4 spots in treated over untreated (CTR) samples. Mean ± SD of three independent experiments performed in triplicate is shown; *P< 0.05, **P< 0.01. (**B**) Representative images of confocal sections (63×) from (A). For each condition, G4 structures (red) and merged images of DAPI counterstained nuclei (blue) with G4s are shown. Scale bar (10 μm) is reported in the images. (C–E) U2OS cells, subjected or not to treatment with the indicated peptides (10 μM for 24 h), were processed for telomeric FISH combined with immunofluorescence analysis of γH2AX, a common marker of DNA damage. (**C**) Histograms showing the percentage of γH2AX-positive cells. (**D**) Quantitative analysis of the percentage of Telomere Induced Foci (TIF)-positive cells. Cells with at least four γH2AX/Telo colocalizations were scored as TIF positive. (**E**) Representative confocal microscopy images (63×) used for the quantitative analyses reported in (C) and (D). γH2AX spots (green), telomere probe spots (red), DAPI stained nuclei (blue) and merged images are shown. For each experimental condition, 4× enlargements of merged fields are reported. Scale bars are shown in the images. (**F**) U2OS cells were treated with 10 μM of R_7_W-Myb^397–415^ and the expression levels of *HER2* and *BCL-2* were evaluated by real-time RT-PCR, at the indicated time-points. Results are expressed as fold change of gene expression in treated cells over their control counterpart (CTR). All the histograms show the mean ± SD of at least three independent experiments performed in triplicate; *P< 0.05, **P< 0.01, ***P< 0.001, ****P< 0.0001.

In the next step, we assessed whether the antitumoral activity of the selected peptides would be dependent on their capability of targeting G4 structures. For this purpose, U2OS cells were subjected to treatment with the different peptides and their ability to promote G4 stabilization was evaluated by immunofluorescence (IF) analyses performed with an antibody, BG4, capable of selectively recognizing these secondary DNA structures (2). As evidenced by the results of confocal analysis, the three peptides promoted a robust and significant increase in the number of G4 structures within cell nuclei (Figure 6A and B), reinforcing the mechanistic idea of a direct correlation between targeting G4 structures and antitumoral activity.

Of note, exposure of cells to G4 ligands can promote an accumulation of DNA damage or, depending on the position of the targeted G4s within the genome, alterations in gene expression (13). Therefore, we aimed to further investigate the mechanism(s) through which the selected peptides drive their G4-mediated antitumoral activity. To address this point, U2OS cells, treated with the conjugated peptides, were first subjected to IF analyses aimed at evaluating the capability of the peptides to induce DNA damage. Interestingly, the IF experiments evidenced significant accumulation of phosphorylated histone H2AX (γH2AX), a well-established marker of DNA damage, following peptide treatment (Figure 6C), with R_7_W-Myb^A400^ eliciting the most pronounced response. Moreover, parallel fluorescence *in-situ* hybridization (FISH) assays demonstrated that γH2AX foci induced by these peptides largely localize at telomeres, as evidenced by formation of the so-called telomere induced foci (TIFs), fluorescent spots deriving from the colocalization of γH2AX with telomeres (Figure 6D and E) (71).

Finally, on the basis of biophysical data showing the ability of these peptides to also bind some gene promoters G4 structures *in vitro*, the R_7_W-conjugated form of the original peptide (R_7_W-Myb^397–415^) was tested for its ability to modulate the expression of *HER2, BCL-2* and *c-KIT*, three important cancer-related genes. Notably, *real-time* RT-PCR time-course analyses performed in U2OS cells revealed undetectable levels of *c-KIT*, indicating that this gene is not relevant in this tumour histotype. Conversely, *HER2* and *BCL-2* were both expressed in the evaluated tumour cells and their levels were found to be transiently modulated by the peptide (Figure 6F). Overall, our results suggest that the observed antitumoral activity of these peptides may mainly pass through their capability of inducing accumulation of DNA damage at the telomeric level.

## Conclusions

G4s play pivotal roles in cancer cell biology, influencing telomere maintenance, transcriptional regulation of cancer-related genes, and genome stability, making them attractive targets for anticancer therapies. To date, numerous synthetic and naturally occurring small molecules have been investigated for their interactions with G4s in the pursuit of discovering potential drug candidates. However, small molecules targeting G4s face significant challenges, such as off-target effects, toxicity, high hydrophobicity, low body clearance, and sometimes even degradation into toxic metabolites (16). Overcoming these limitations is crucial for developing effective G4-targeting drugs, requiring molecules that distinguish G4 from duplex DNA, exhibit low off-target effects, possess cell-penetrating capabilities, and avoid degradation into toxic metabolites in cell. Despite extensive research, a molecule that meets all these criteria has yet to be discovered.

Several proteins interact with DNA G4s (9,72,73), prompting researchers to explore the potential therapeutic use of G4-binding proteins. However, challenges such as high manufacturing costs, unsuitability for oral administration due to gut degradation, and inefficient penetration to reach target sites due to their large size seem to prevent their widespread use.

Biologically active peptides may represent promising alternatives to small molecules and whole proteins (74,75). An unresolved question is whether the DNA recognition motif of proteins can be exploited to derive peptides that selectively target G4s. Intrigued by this question, in this study we analysed the interaction between a peptide derived from the G4-binding domain of the yeast protein Rap1 (Myb^397–415^) and various biologically relevant G4 DNA structures with different topologies.

CD and MST experiments showed that Myb^397–415^ is able to bind to and stabilize most G4s, exhibiting particularly strong affinity for parallel G4 motifs (with *c-KIT2*, *HER2* and *HT-FANA* G4s resulting the best targets of the series). Furthermore, CD spectra analysis indicated no structural rearrangements or unfolding of the G4s upon peptide binding. Interestingly, the negligible interaction observed between Myb^397–415^ and the duplex model *ds_12_* confirmed that the binding of the peptide to DNA is not solely mediated by electrostatic interactions and suggested that there is a specific affinity for G4 structures. UVRR analysis indicated that the interaction of Myb^397–415^ primarily involves the external G-tetrads, as evidenced by perturbations observed in the guanine bands upon binding. The ITC analysis provided insights into the thermodynamics of G4–peptide interactions, revealing an enthalpy-driven binding process in all cases, accompanied by an unfavourable entropic contribution. The absence of observable structural changes in the G4s suggests that upon DNA binding, the peptide may undergo structural changes from its initially random conformation in solution, a hypothesis supported by CD data.

Through the alanine scanning approach, we systematically assessed the influence of each residue on the peptide's ability to bind to and stabilize G4-forming DNA. This enabled us to discern the key and less important amino acids in the interaction with G4 DNA, ultimately leading to the identification of two novel peptides with enhanced G4-binding properties compared to the original one.

Surprisingly, despite being excellent G4 ligands *in vitro*, Myb^397–415^ and its most promising derivatives (Myb^A400^ and Myb^A412^) did not significantly affect cancer cell viability due to their inability to effectively cross the cell membrane. The development of CPP-conjugated peptides allowed us to obtain derivatives with significant cytotoxic effects on cancer cells. Interestingly, the conjugated peptides were able to exert a potent anticancer activity across various tumour histotypes through a G4 dependent mechanism, independently from the molecular mechanisms of telomere maintenance and DNA repair.

Overall, this study establishes the potentiality of this class of peptides to target G4 structures and their anticancer activity. The results obtained will undoubtedly contribute to the development of other derivatives with the aim of further improving peptide affinity and selectivity for G4 structures. The data collected could pave the way for the development of new therapeutic approaches capable of leveraging the advantages of therapeutic biomolecules with the anti-tumour properties of canonical G4-ligands.

## Supplementary Material

gkae471_Supplemental_File

## Data Availability

All data are available from the corresponding authors upon request.
