# Supplementary material for: Unlocking the potential of protein-derived peptides to target G-quadruplex DNA: from recognition to anticancer activity
Source: Nucleic Acids Res. 2024 Jun 3;52(12):6748–62. doi: 10.1093/nar/gkae471 (PMC11229374; doi:10.1093/nar/gkae471)
Supplement: gkae471_Supplemental_File [file gkae471_supplemental_file.pdf]

SUPPLEMENTARY MATERIAL

Unlocking the potential of protein-derived peptides to target G-quadruplex DNA: From recognition to anticancer activity

Francesco Merlino[1,†], Simona Marzano[1,†], Pasquale Zizza[2,*], Federica D'Aria[1], Nicola Grasso[1], Alice Carachino[2], Sara Iachettini[2], Annamaria Biroccio[2], Silvia Di Fonzo[3], Paolo Grieco[1], Antonio Randazzo[1], Jussara Amato[1,*], Bruno Pagano[1,*]

[1] Department of Pharmacy, University of Naples Federico II, Naples, 80131, Italy

[2] Oncogenomic and Epigenetic Unit, IRCCS-Regina Elena National Cancer Institute, Rome, 00144, Italy

[3] Elettra-Sincrotrone Trieste S. C. p. A., Science Park, Trieste, 34149, Italy

* To whom correspondence should be addressed. Tel: +39 081678641; E-mail: bruno.pagano@unina.it

Correspondence may also be addressed to Jussara Amato. E-mail: jussara.amato@unina.it

Correspondence may also be addressed to Pasquale Zizza. E-mail: pasquale.zizza@ifo.it

† The first two authors should be regarded as Joint First Authors.

Detailed synthetic procedure to yield Myb$^{397-415}$ peptide and its derivatives

Rink amide resin (0.2 mmol for each sequence; 0.63 mmol/g as loading, 100-200 mesh as particle size) was placed in a 10 mL polypropylene syringe tube (ISOLUTE® SPE filtration column by Biotage, Uppsala, Sweden), equipped with Teflon® filter, stopper, and stopcock, and pre-swollen in DMF on an automated shaker (Multi Reax vortexer from Heidolph Instruments GmbH, Schwabach, Germany) at rt for 30 min. Since the resin was stored as protected by Fmoc, it was treated with 20% piperidine in DMF solution (5 min × 1; 25 min × 1). Then coupling with the first N^{α}-Fmoc-protected amino acid (3 equiv) was achieved using HBTU/HOBt (3 equiv each) and DIEA (6 equiv), on an automated shaker for 2 h, at rt. At this point the synthesis continued according to the US-SPPS method. To remove the Fmoc group, a 20% piperidine in DMF solution was added to the resin and the tube reactor was then placed in an ultrasonic bath (Sonorex RK 52 H from Bandelin Electronic, Germany) (0.5 + 1 min) with the reaction mixture not exceeding to the water level. The couplings were carried out by treating with a solution of the N^{α}-Fmoc-protected amino acid (3 equiv), COMU (3 equiv), Oxyma (3 equiv), and DIEA (6 equiv) in DMF and exposing the resin to the ultrasonic irradiation for 10 min. After each Fmoc-deprotection and coupling step, filtering and washing of the resin were performed with both DMF (3 × 2 mL) and DCM (3 × 2 mL). During the construction of the target resin-bound linear peptides, the amino group in the *N*-terminus was treated differently depending on the series of derivatives. Regarding Myb$^{397-415}$ and Ala-mutated derivatives (Myb^{A397}-Myb^{A415}) (Table S1), the *N*-terminal primary amine was acetylated by treatment with a solution of Ac_2O (2 equiv) and DIPEA (4 equiv) in DMF, and the reactor was placed on an automated shaker for 30 min at rt. While, in the construction of FITC-labeled peptides (FITC-Myb$^{397-415}$, FITC-Myb^{A400}, and FITC-Myb^{A412}) (Table S1) the *N*-terminal primary amine was treated with fluorescein isothiocyanate (2 equiv) and trietylamine (5 equiv) up to 16 h at rt, after introduction of a spacer, such as O2Oc, through a coupling reaction with Fmoc-O2Oc and subsequent removal of the Fmoc group, both performed as described above. Otherwise, CPP-conjugated peptides (Table S1) retain an unmodified primary amino function in that position.

All resin-bound linear peptides were dried under *vacuum* and finally cleaved by treatment with a cocktail of TFA/TIS/H_2O (95:2.5:2.5, *v/v/v*) on a magnetic stirrer plate (200 rpm) for 3 h, at rt. Crude peptides were recovered by precipitation with chilled anhydrous Et_2O and then centrifuged (6000 rpm × 15 min). The supernatants were carefully removed and the resulting white to light beige-colored amorphous solids were dried and dissolved in water/acetonitrile (9:1, *v/v*) to be analyzed by reversed-phase HPLC.

Purification of peptides was performed by RP-HPLC (Shimadzu Preparative Liquid Chromatograph LC-8A) equipped with a preparative column (Phenomenex Kinetex XB-C18 column, 5 µm, 100 Å, 150 × 21.2 mm) using linear gradients of MeCN (0.1% TFA) in water (0.1% TFA), from 10 to 60% over 30 min, with a flow rate of 10 mL/min and UV detection at 220 nm. The final products were obtained by freeze-drying the appropriate fractions after removal of MeCN by rotary evaporation. Analytical UHPLC (Shimadzu Nexera Liquid Chromatograph LC-30AD/DGU-20A$_{5R}$/SIL-30AC/SPD-M20A/CTO-20AC/CBM-20A) was used to assess the purity of peptides and analyses were performed on a Phenomenex Kinetex reversed-phase column (XB-C18, 5 µm, 100 Å, 150 × 4.6 mm) with a flow rate of 1 mL/min using a gradient of MeCN (0.1% TFA) in water (0.1% TFA), from 10 to 90% over 15 min, and UV detection at 220 nm.

Table S1. Name, sequence, and analytical data of the synthesized peptides.

Name	Sequence (Ac → NH_2)	t_R[a] (min)	Molecular Formula	$[M+H]^+_{calc}$	$[(M+nH)/n]^+_{obs}$
Myb[397-415]	NHTGNSIRHRFRVYLSKRL	10.5	$C_{105}H_{172}N_{39}O_{26}^+$	2395.7775	799.4483, n= 3<br>599.8384, n= 4<br>480.0723, n= 5
Myb[A397]	**A**HTGNSIRHRFRVYLSKRL	10.5	$C_{104}H_{171}N_{38}O_{25}^+$	2353.7525	785.1134, n= 3<br>589.0874, n= 4<br>471.4716, n= 5
Myb[A398]	N**A**TGNSIRHRFRVYLSKRL	10.8	$C_{102}H_{170}N_{37}O_{26}^+$	2330.7145	777.4421, n= 3<br>583.3332, n= 4<br>466.8684, n= 5
Myb[A399]	NH**A**GNSIRHRFRVYLSKRL	10.2	$C_{104}H_{170}N_{39}O_{25}^+$	2366.7515	789.4458, n= 3<br>592.3364, n= 4<br>474.0706, n= 5
Myb[A400]	NHT**A**NSIRHRFRVYLSKRL	10.3	$C_{106}H_{174}N_{39}O_{26}^+$	2410.8045	804.1268, n= 3<br>603.3482, n= 4<br>482.8809, n= 5
Myb[A401]	NHTG**A**SIRHRFRVYLSKRL	10.2	$C_{104}H_{171}N_{38}O_{25}^+$	2353.7525	785.1136, n= 3<br>589.0874, n= 4<br>471.4714, n= 5
Myb[A402]	NHTGN**A**IRHRFRVYLSKRL	10.5	$C_{105}H_{172}N_{39}O_{25}^+$	2380.7785	794.1185, n= 3<br>595.8405, n= 4<br>476.8739, n= 5
Myb[A403]	NHTGNS**A**RHRFRVYLSKRL	10.1	$C_{102}H_{166}N_{39}O_{26}^+$	2354.6965	785.7684, n= 3<br>589.3274, n= 4<br>471.6634, n= 5
Myb[A404]	NHTGNSI**A**HRFRVYLSKRL	10.7	$C_{102}H_{165}N_{36}O_{26}^+$	2311.6675	771.0942, n= 3<br>578.5726, n= 4<br>463.0599, n= 5
Myb[A405]	NHTGNSIR**A**RFRVYLSKRL	10.7	$C_{102}H_{170}N_{37}O_{26}^+$	2330.7145	777.4406, n= 3<br>583.3334, n= 4<br>466.8684, n= 5
Myb[A406]	NHTGNSIRH**A**FRVYLSKRL	10.9	$C_{102}H_{165}N_{36}O_{26}^+$	2311.6675	771.4297, n= 3<br>578.8231, n= 4<br>463.4482, n= 5
Myb[A407]	NHTGNSIRHR**A**RVYLSKRL	9.7	$C_{99}H_{168}N_{39}O_{26}^+$	2320.6795	774.1054, n= 3<br>580.8310, n= 4<br>464.8663, n= 5
Myb[A408]	NHTGNSIRHRF**A**VYLSKRL	10.8	$C_{102}H_{165}N_{36}O_{26}^+$	2311.6675	771.0964, n= 3<br>578.5733, n= 4<br>463.0606, n= 5
Myb[A409]	NHTGNSIRHRFR**A**YLSKRL	10.4	$C_{103}H_{168}N_{39}O_{26}^+$	2368.7235	790.1063, n= 3<br>592.8315, n= 4<br>474.4668, n= 5
Myb[A410]	NHTGNSIRHRFRV**A**LSKRL	10.2	$C_{99}H_{168}N_{39}O_{25}^+$	2304.6805	769.1077, n= 3<br>577.0828, n= 4<br>461.8681, n= 5
Myb[A411]	NHTGNSIRHRFRVY**A**SKRL	9.5	$C_{102}H_{166}N_{39}O_{26}^+$	2354.6965	785.4327, n= 3<br>589.3272, n= 4<br>471.6632, n= 5
Myb[A412]	NHTGNSIRHRFRVYL**A**KRL	10.4	$C_{105}H_{172}N_{39}O_{25}^+$	2380.7785	794.1177, n= 3<br>595.8401, n= 4<br>476.8740, n= 5
Myb[A413]	NHTGNSIRHRFRVYLS**A**RL	10.5	$C_{102}H_{165}N_{38}O_{26}^+$	2339.6815	780.4296, n= 3<br>585.5744, n= 4<br>468.6612, n= 5
Myb[A414]	NHTGNSIRHRFRVYLSK**A**L	11.0	$C_{102}H_{165}N_{36}O_{26}^+$	2311.6675	771.0945, n= 3<br>578.5728, n= 4<br>463.0600, n= 5
Myb[A415]	NHTGNSIRHRFRVYLSKR**A**	10.3	$C_{102}H_{166}N_{39}O_{26}^+$	2354.6965	785.4333, n= 3<br>589.3272, n= 4<br>471.6632, n= 5
FITC-Myb[397-415]	FITC-O2Oc-NHTGNSIRHRFRVYLSKRL	11.2	$C_{130}H_{194}N_{41}O_{33}S^+$	2891.2955	963.4814, n= 3<br>723.6122, n= 4<br>579.2920, n= 5

FITC-Myb^{A400}	FITC-O2Oc-NHT**A**NSIRHRFRVYLSKRL	11.3	$C_{131}H_{196}N_{41}O_{33}S^{+}$	2905.3225	969.7358, n= 3<br>726.6183, n= 4<br>581.8972, n= 5
FITC-Myb^{A412}	FITC-O2Oc-NHTGNSIRHRFRVYL**A**KRL	11.2	$C_{130}H_{194}N_{41}O_{32}S^{+}$	2875.2965	959.6982, n= 3<br>719.6278, n= 4<br>576.0973, n= 5
Tat-Myb$^{397-415}$	*RKKRRQRRR*-GG-NHTGNSIRHRFRVYLSKRL	10.1	$C_{160}H_{280}N_{71}O_{37}^{+}$	3790.4595	948.8591, n= 4<br>759.6100, n= 5
YG-Myb$^{397-415}$	*YGRKKRRQRRR*-GG-NHTGNSIRHRFRVYLSKRL	9.7	$C_{171}H_{292}N_{73}O_{40}^{+}$	4010.6875	1003.9075, n= 4<br>803.6386, n= 5
R_6W_3-Myb$^{397-415}$	*RRWWRRWRR*-GG-NHTGNSIRHRFRVYLSKRL	10.3	$C_{176}H_{278}N_{71}O_{36}^{+}$	3964.6205	991.9150, n= 4<br>793.7056, n= 5
R_7W-Myb$^{397-415}$	*RRRRRRRW*-GG-NHTGNSIRHRFRVYLSKRL	9.9	$C_{160}H_{270}N_{71}O_{35}^{+}$	3748.3815	938.7639, n= 4<br>750.3069, n= 5
R_7W-Myb^{A400}	*RRRRRRRW*-GG-NHT**A**NSIRHRFRVYLSKRL	10.3	$C_{161}H_{272}N_{71}O_{35}^{+}$	3762.4085	941.7808, n= 4<br>753.5483, n= 5
R_7W-Myb^{A412}	*RRRRRRRW*-GG-NHTGNSIRHRFRVYL**A**KRL	10.3	$C_{160}H_{270}N_{71}O_{34}^{+}$	3732.3825	934.8015, n= 4<br>746.6064, n= 5

[a] Retention time extrapolated from the corresponding HPLC chromatograms shown in Figures S1-29.

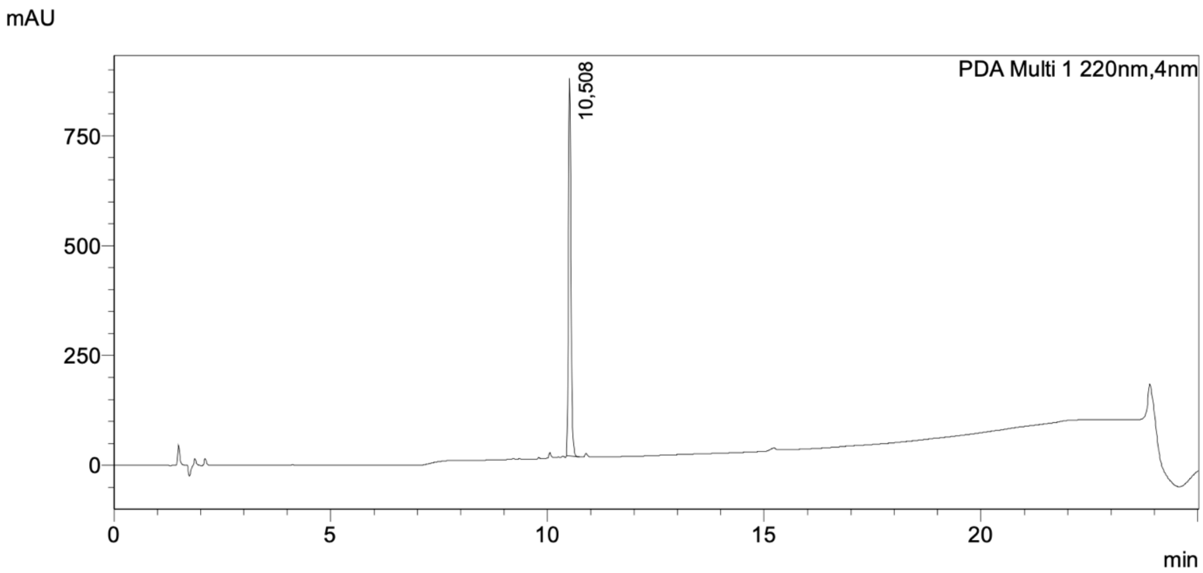

Figure S1. Chromatogram of Myb$^{397-415}$ (t_R = 10.5 min).

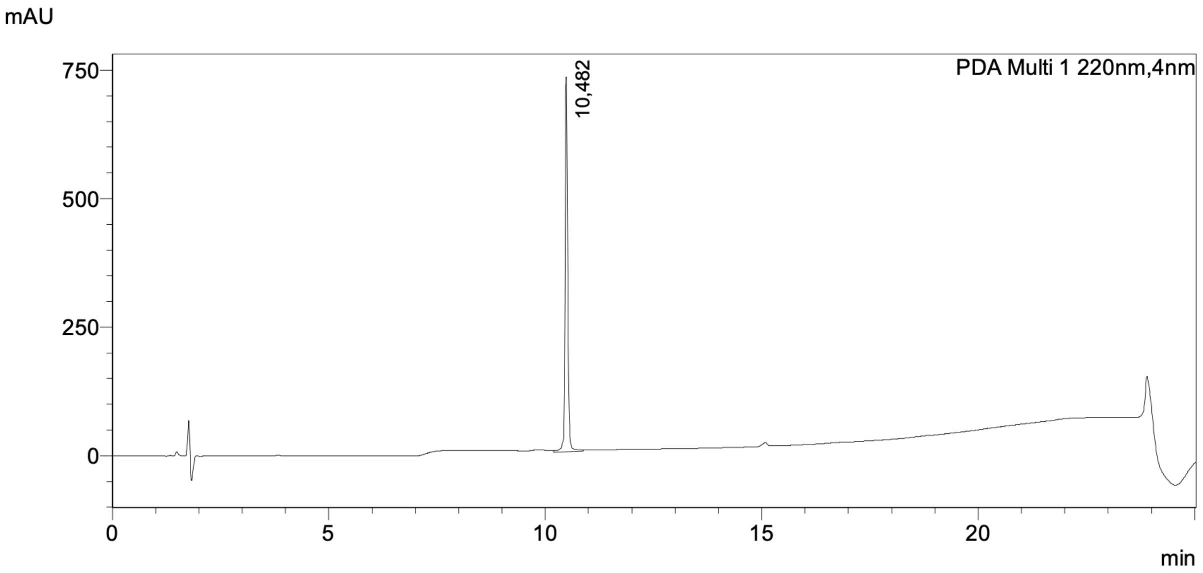

Figure S2. Chromatogram of Myb^{A397} (t_R = 10.5 min).

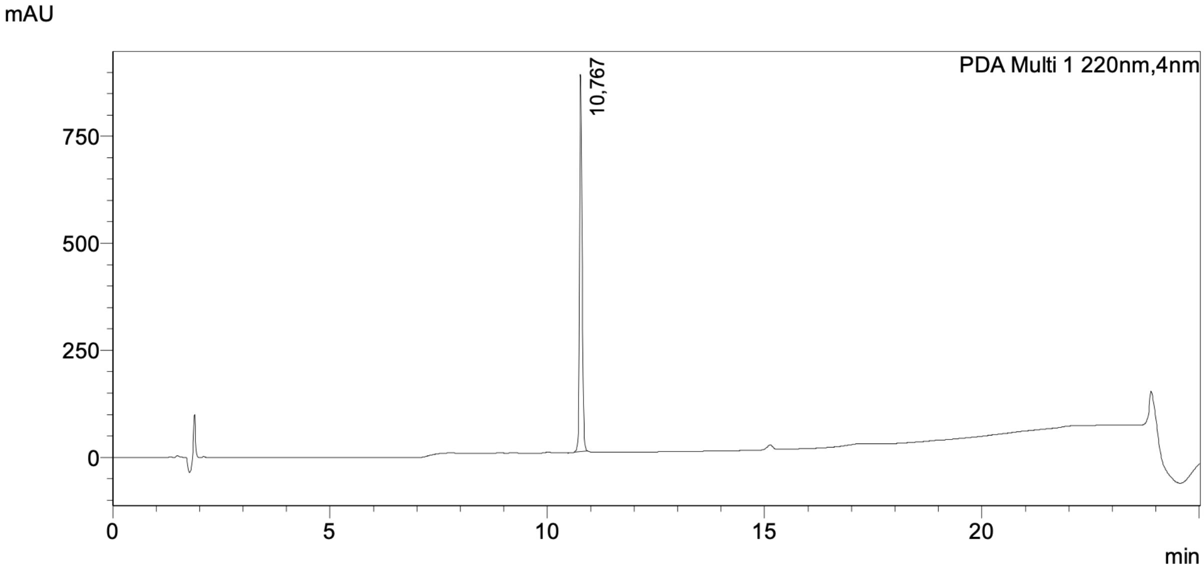

Figure S3. Chromatogram of Myb^{A398} (t_R = 10.8 min).

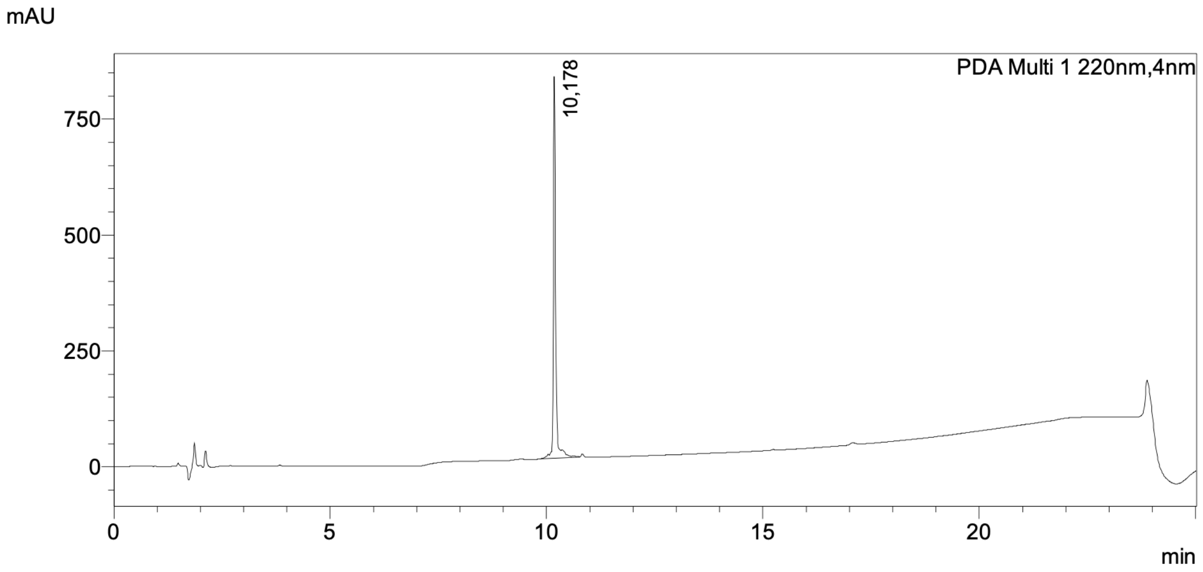

Figure S4. Chromatogram of Myb^{A399} (t_R = 10.2 min).

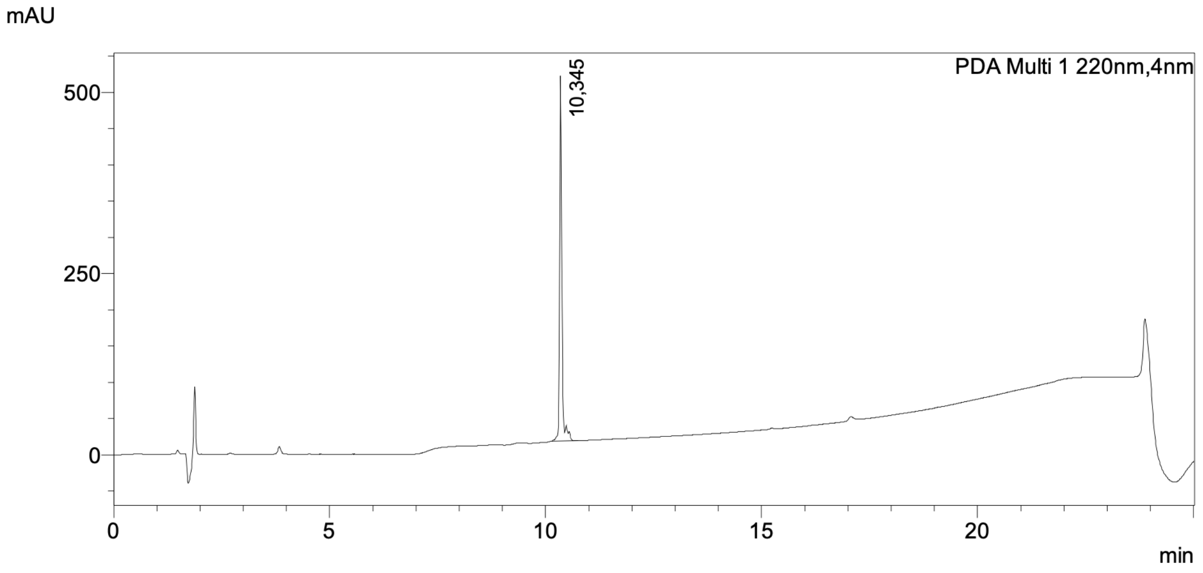

Figure S5. Chromatogram of Myb^{A400} (t_R = 10.3 min).

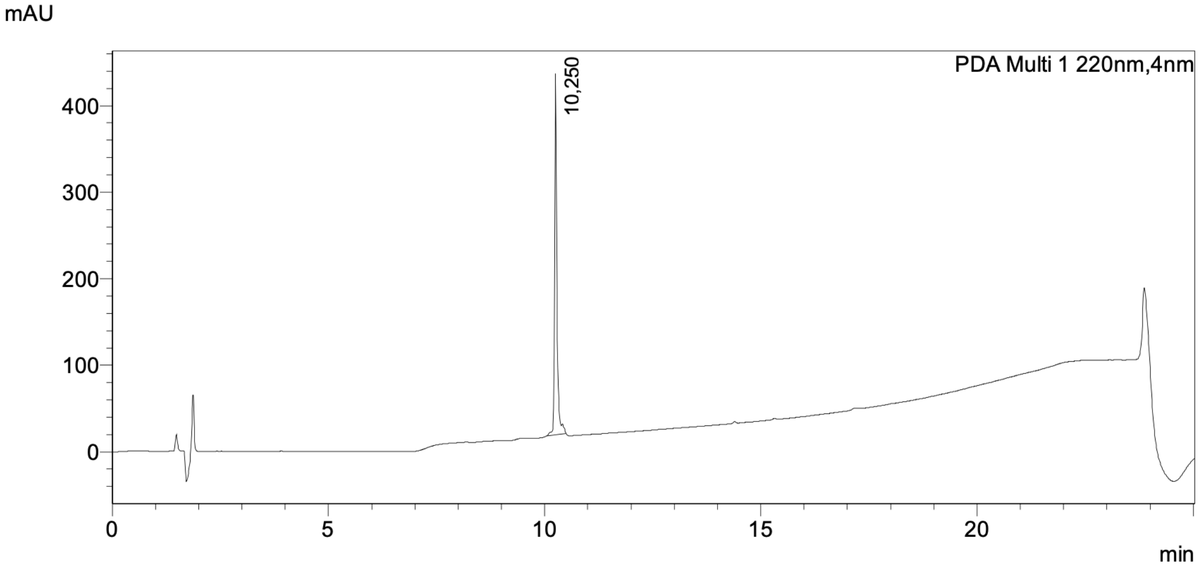

Figure S6. Chromatogram of Myb^{A401} (t_R = 10.2 min).

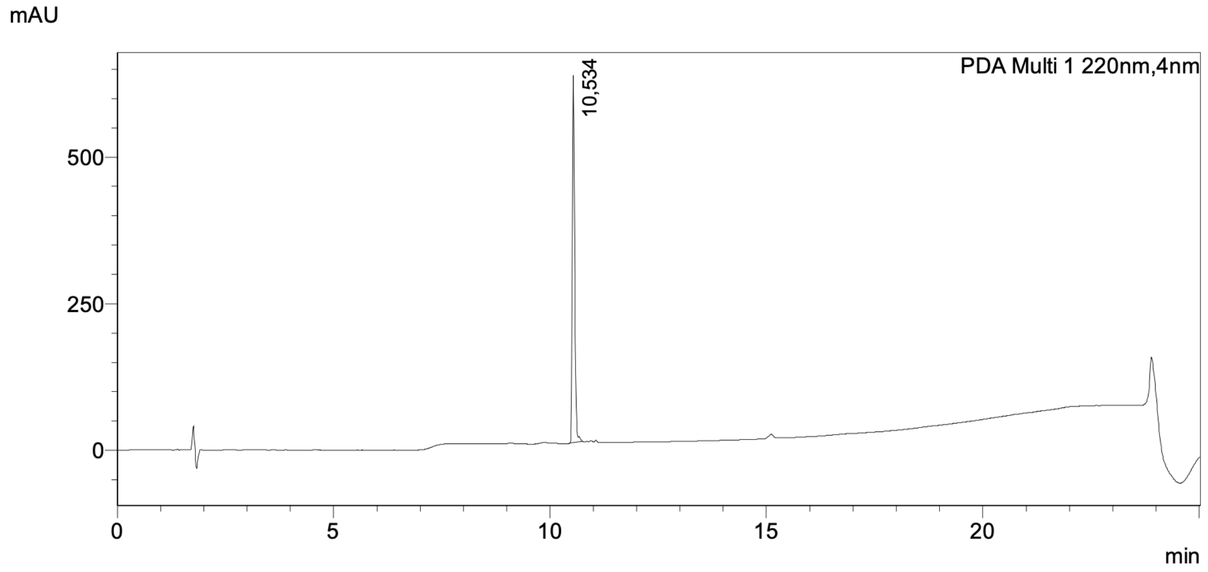

Figure S7. Chromatogram of Myb^{A402} (t_R = 10.5 min).

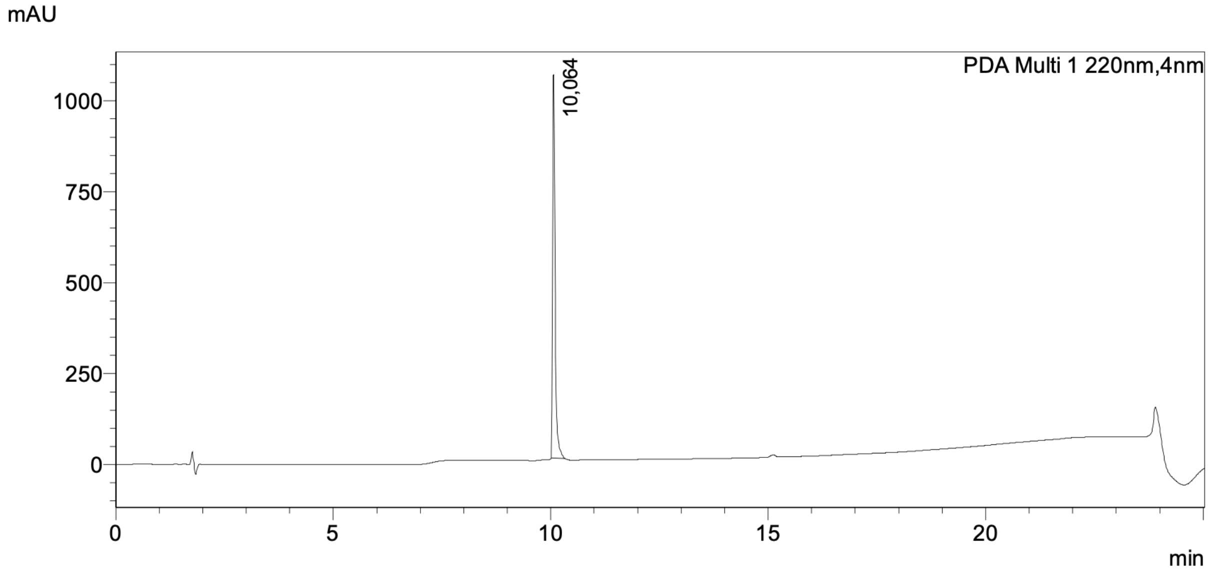

Figure S8. Chromatogram of Myb^{A403} (t_R = 10.1 min).

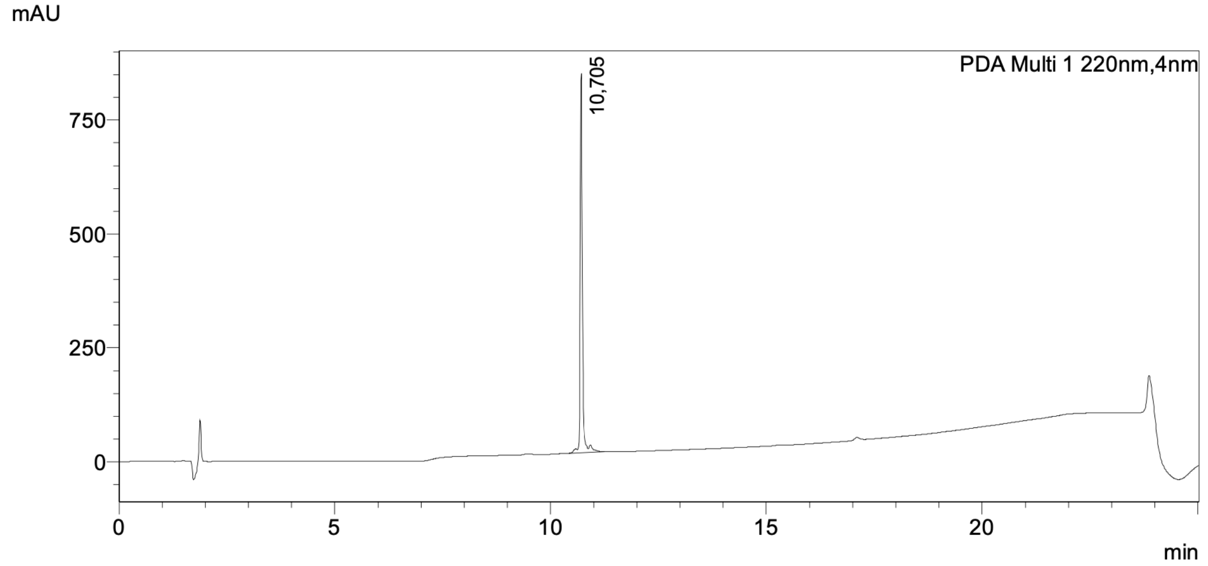

Figure S9. Chromatogram of Myb^{A404} (t_R = 10.7 min).

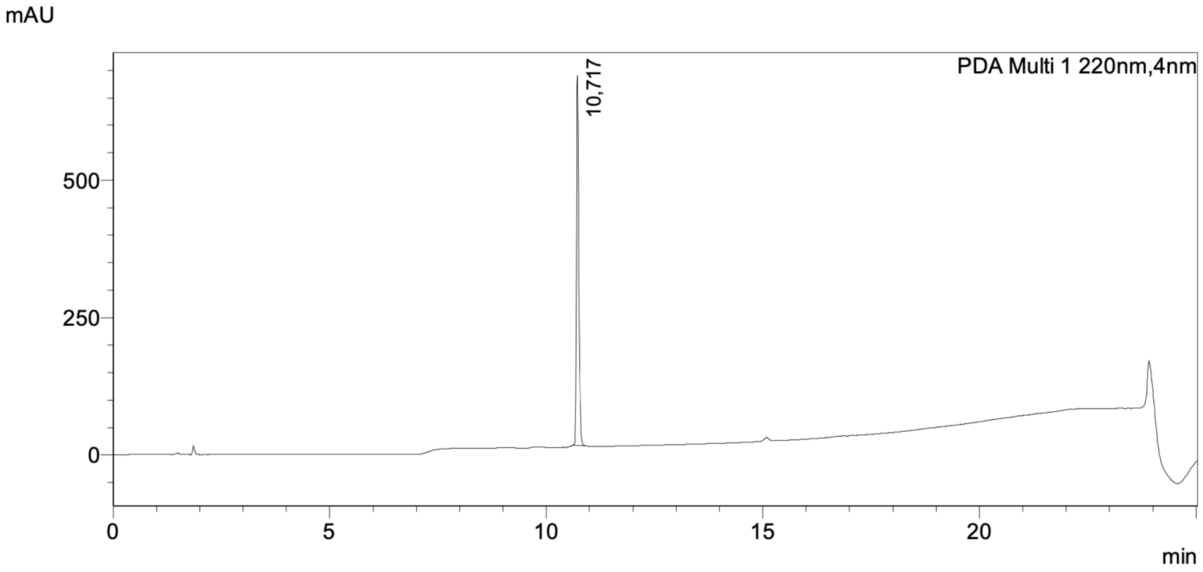

Figure S10. Chromatogram of Myb^{A405} (t_R = 10.7 min).

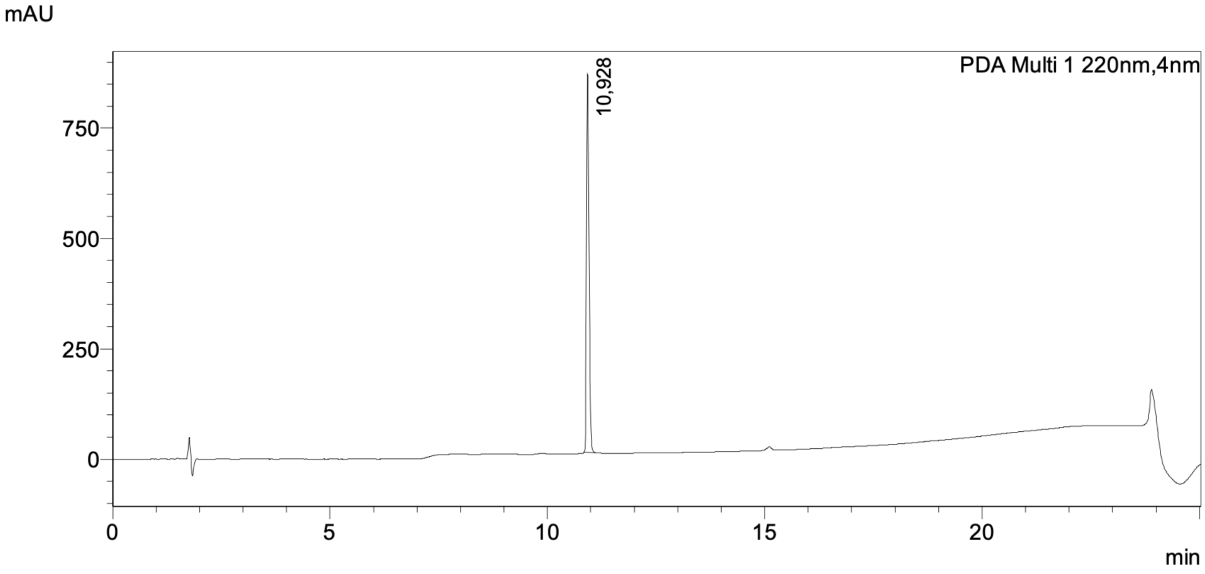

Figure S11. Chromatogram of Myb^{A406} (t_R = 10.9 min).

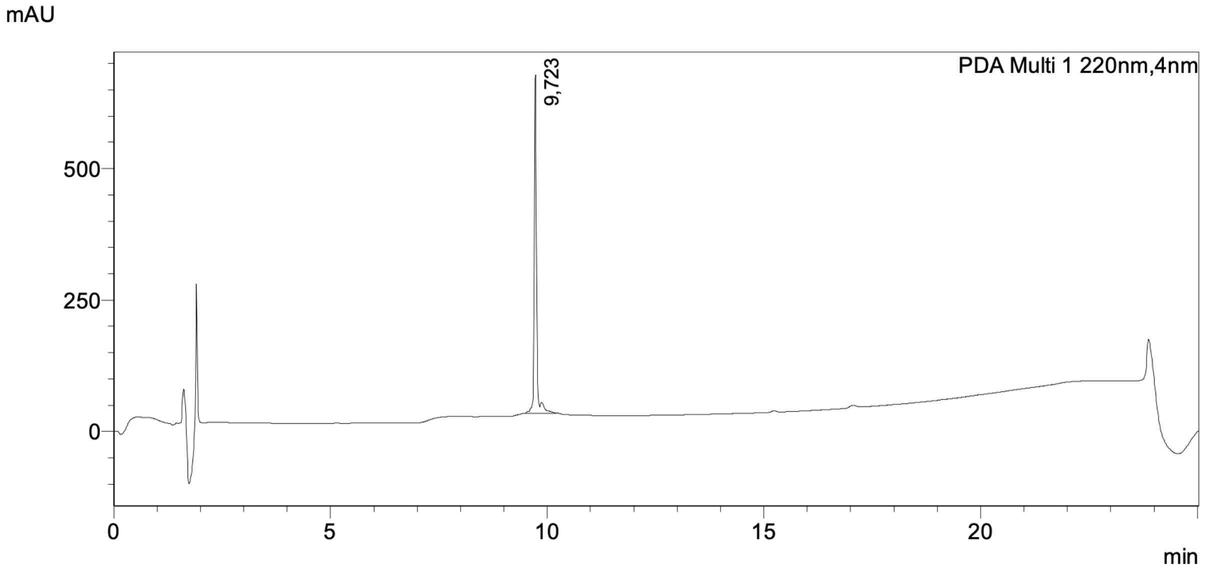

Figure S12. Chromatogram of Myb^{A407} (t_R = 9.7 min).

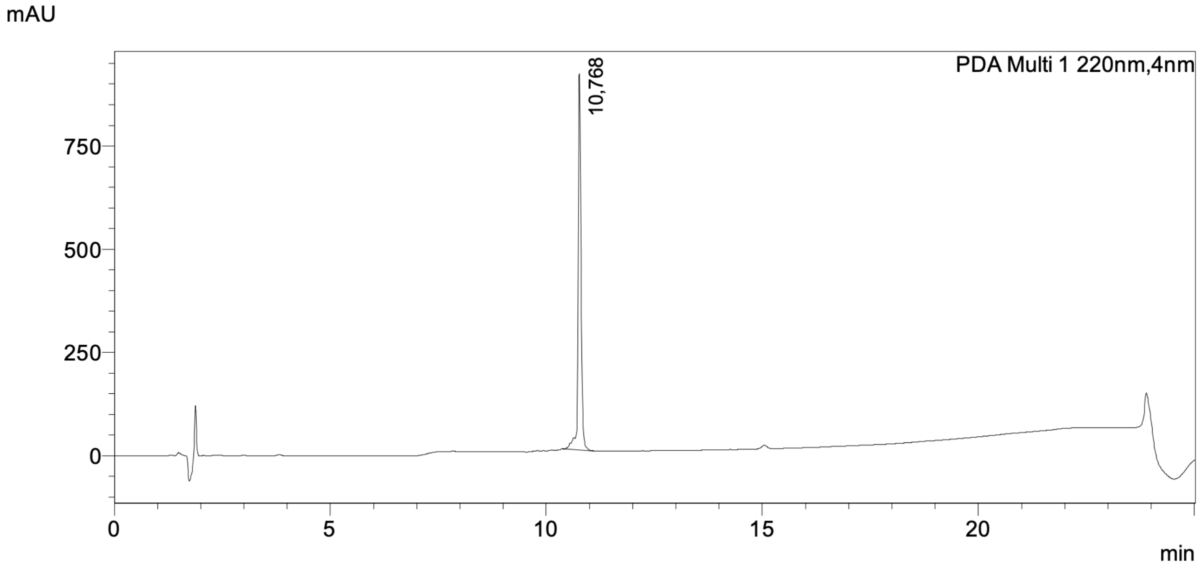

Figure S13. Chromatogram of Myb^{A408} (t_R = 10.8 min).

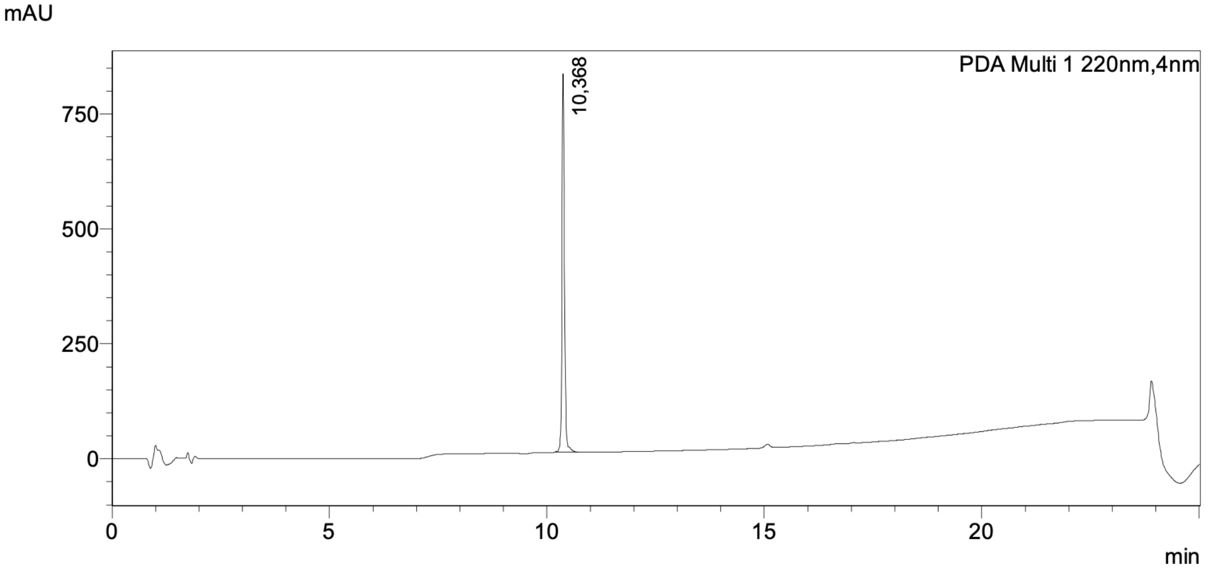

Figure S14. Chromatogram of Myb^{A409} (t_R = 10.4 min).

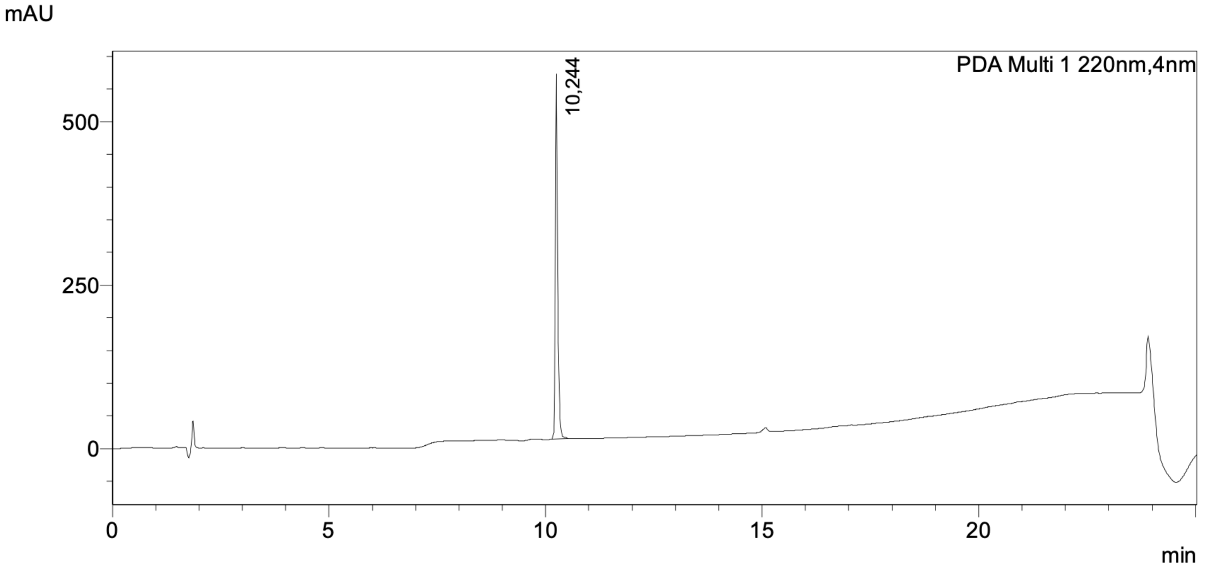

Figure S15. Chromatogram of Myb^{A410} (t_R = 10.2 min).

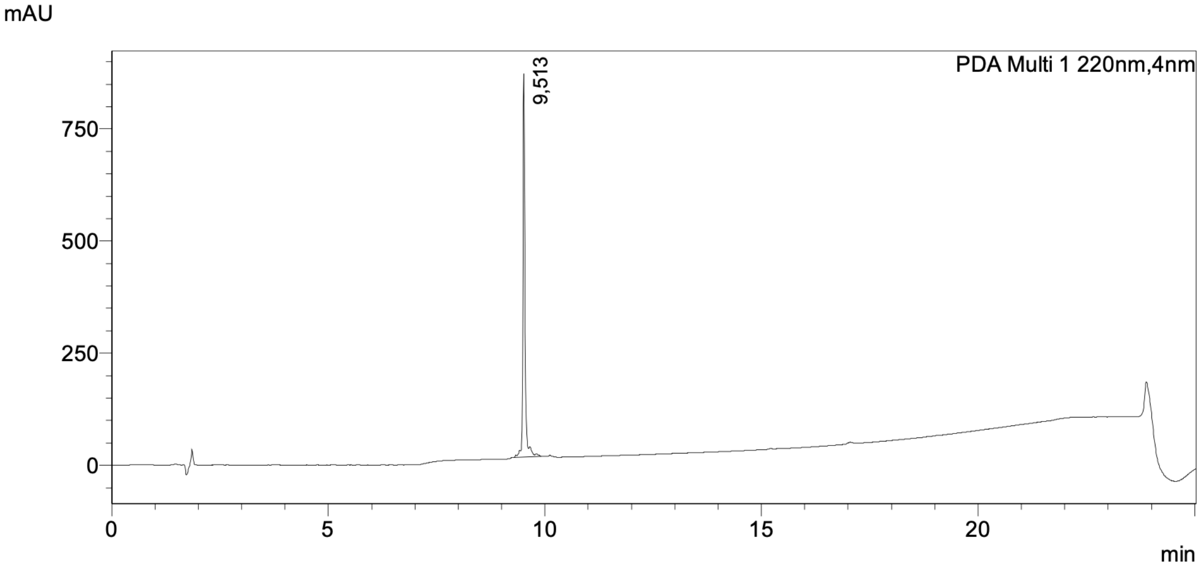

Figure S16. Chromatogram of Myb^{A411} (t_R = 9.5 min).

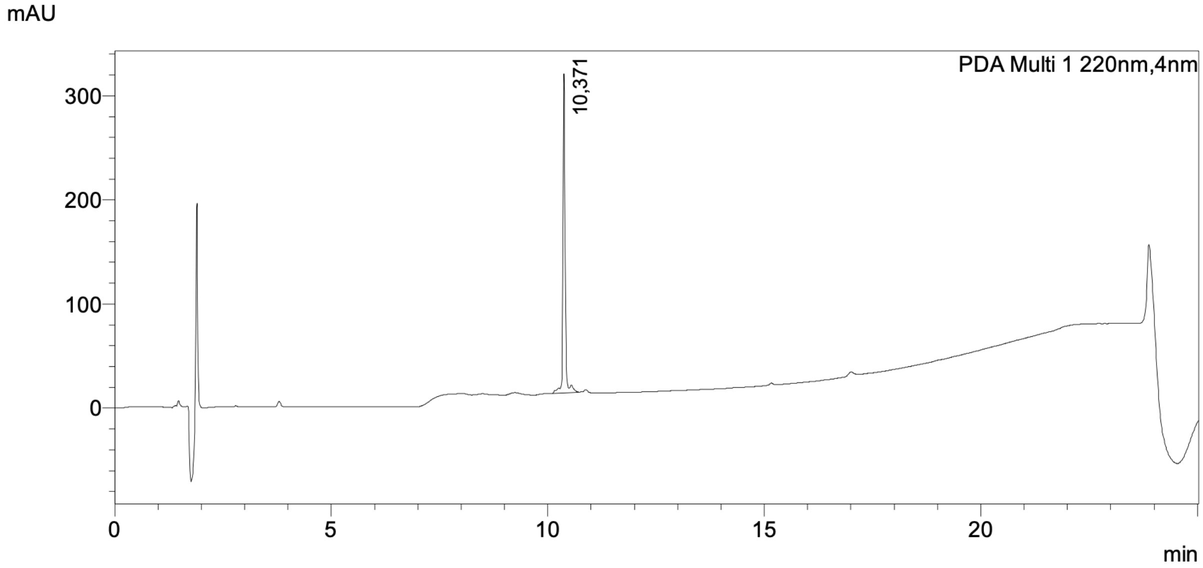

Figure S17. Chromatogram of Myb^{A412} (t_R = 10.4 min).

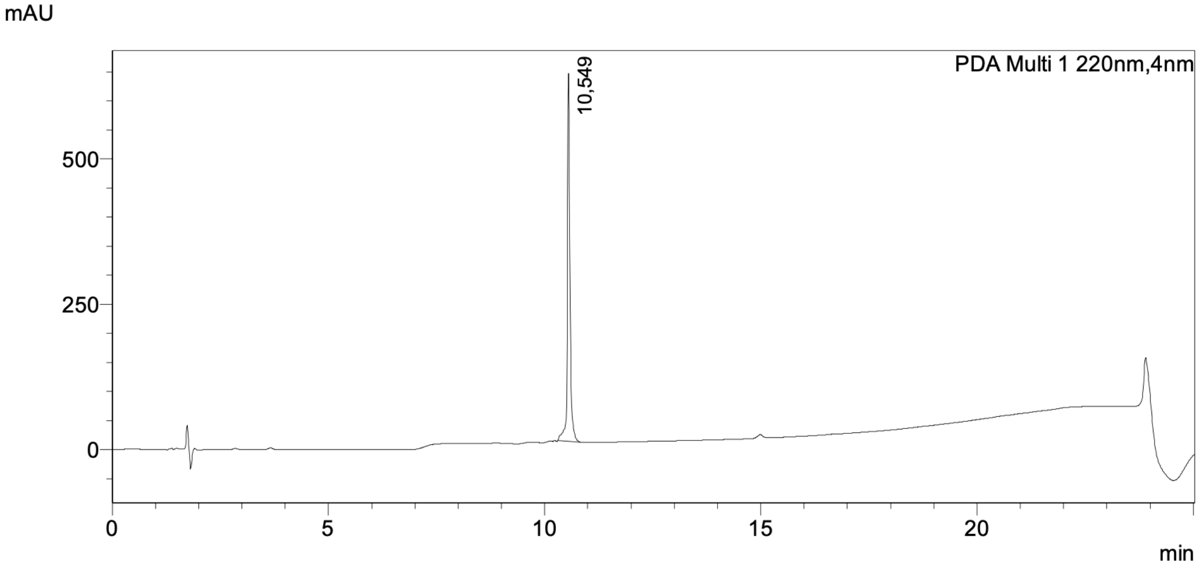

Figure S18. Chromatogram of Myb^{A413} (t_R = 10.5 min).

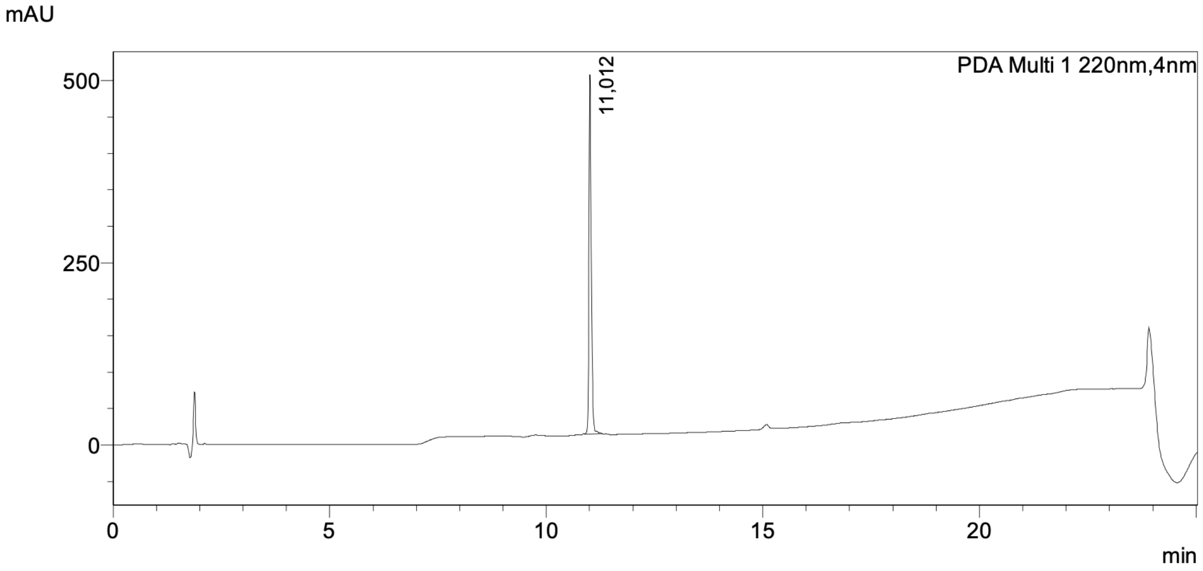

Figure S19. Chromatogram of Myb^{A414} (t_R = 11.0 min).

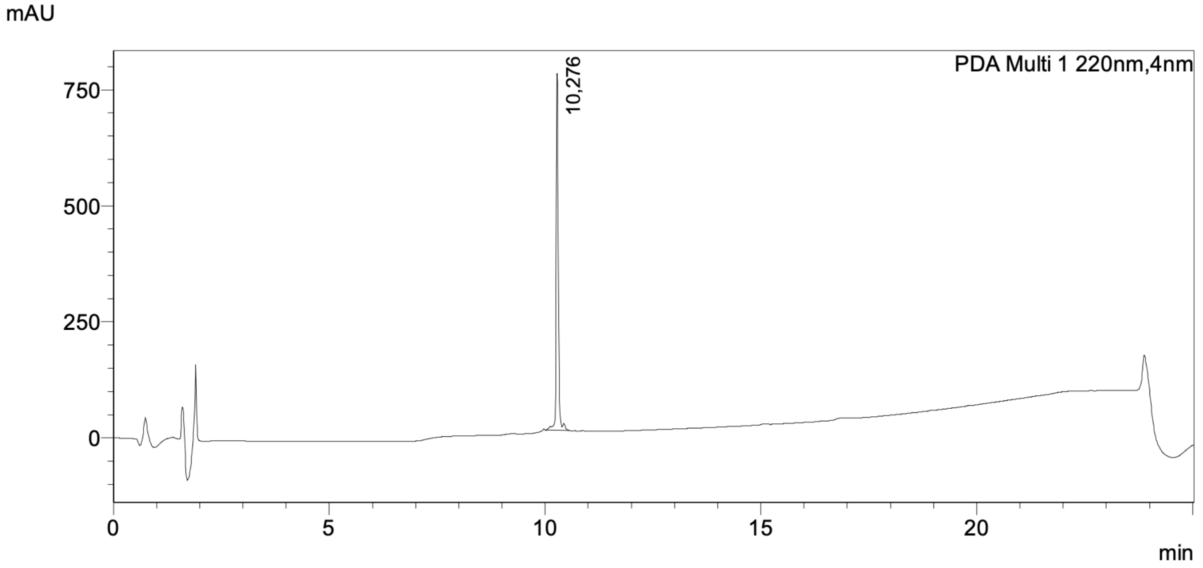

Figure S20. Chromatogram of Myb^{A415} (t_R = 10.3 min).

a)

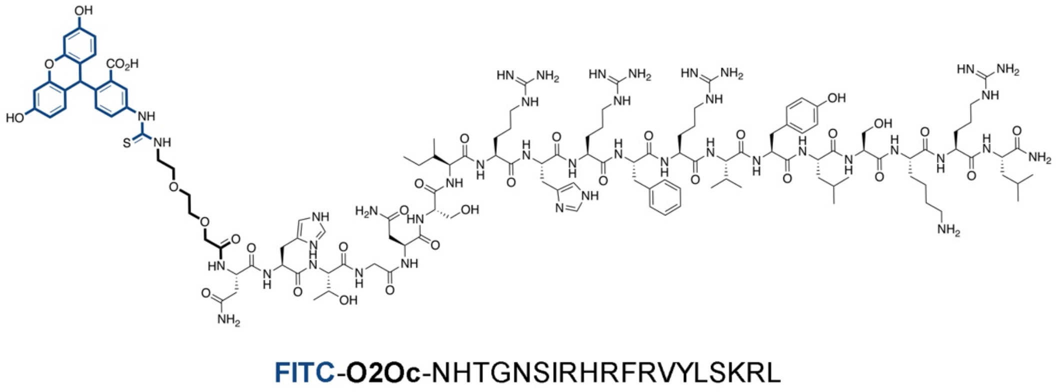

b)

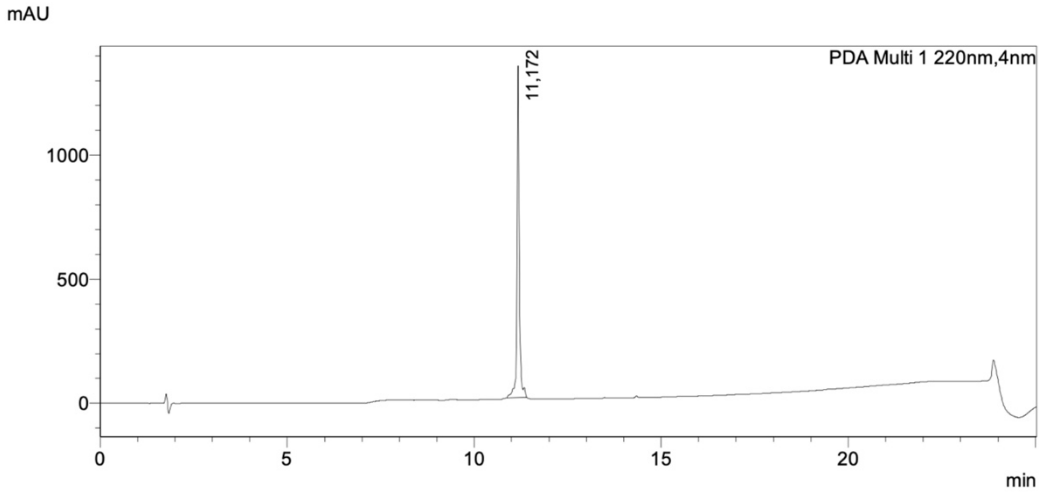

c)

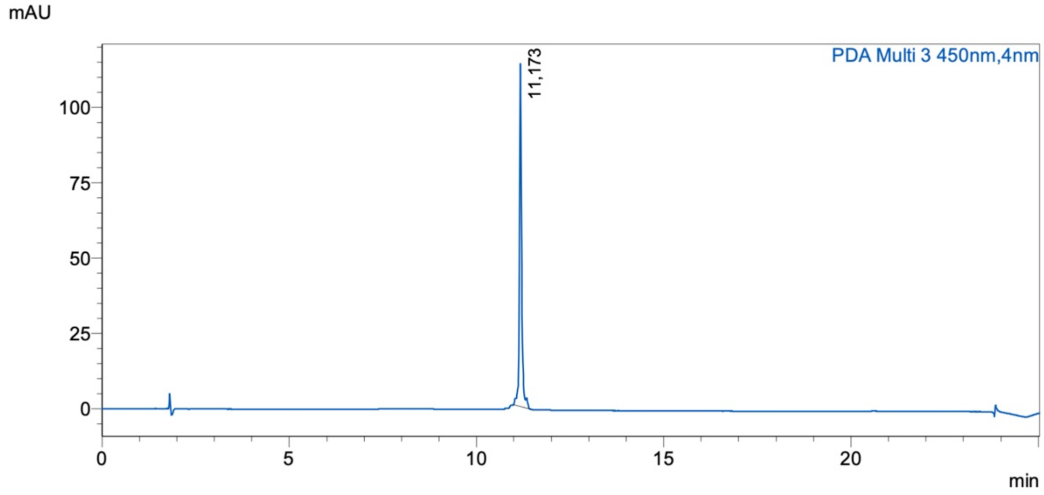

Figure S21. Structure (a) and HPLC chromatograms of FITC-Myb[397-415] (t_R = 11.2 min) acquired by an analytical UHPLC and monitored by UV detection at 220 (b) and 450 (c) nm.

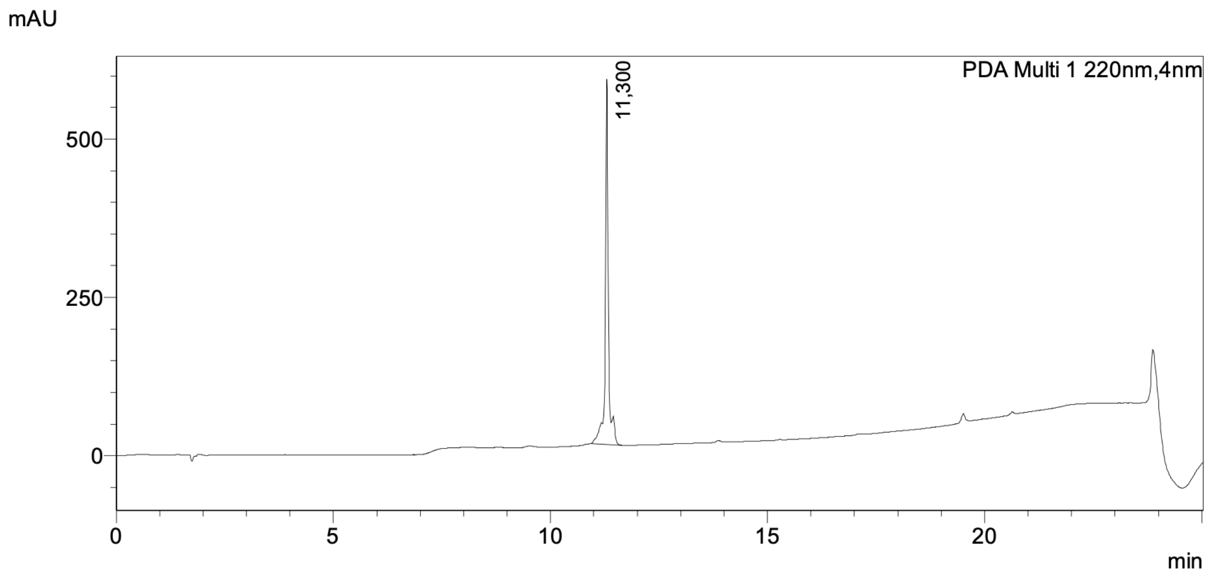

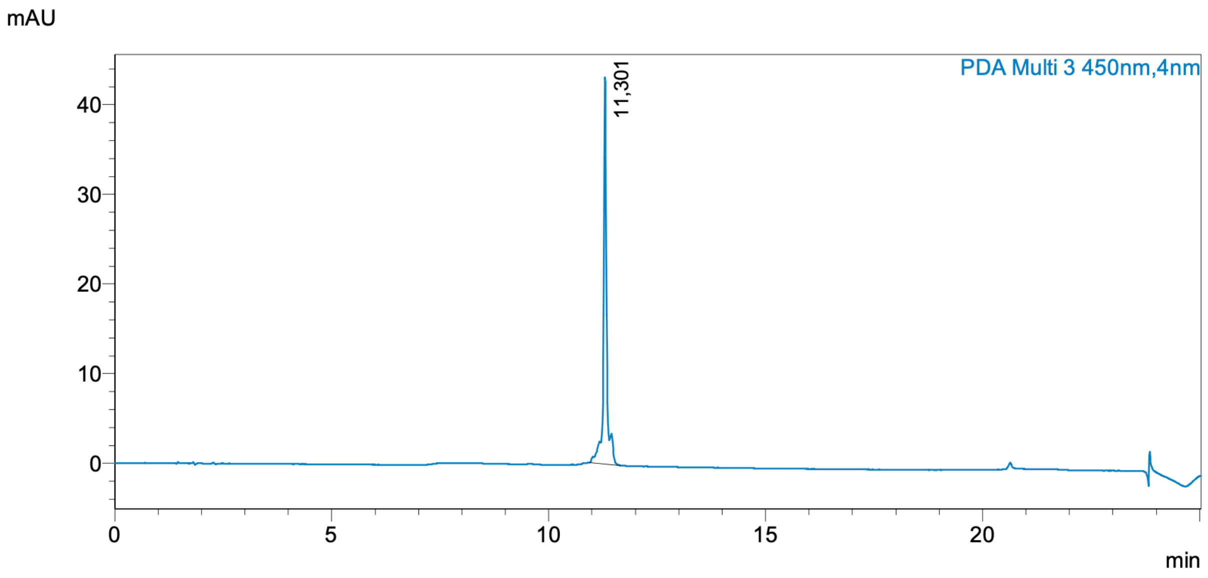

Figure S22. HPLC chromatograms of FITC-Myb^{A400} (t_R = 11.3 min) acquired by an analytical UHPLC and monitored by UV detection at 220 and 450 nm.

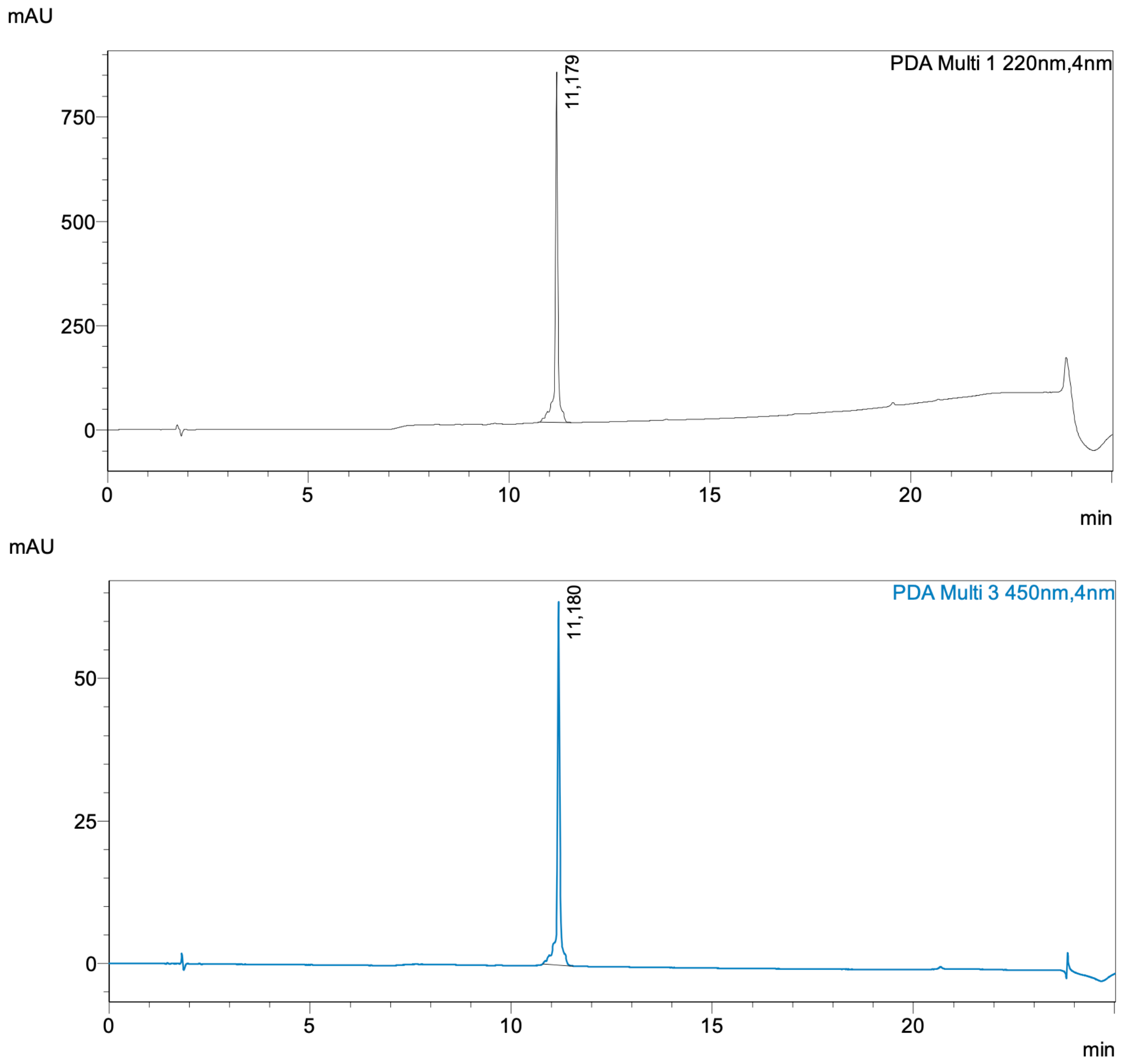

Figure S23. HPLC chromatograms of FITC-Myb^{A412} (t_R = 11.2 min) acquired by an analytical UHPLC and monitored by UV detection at 220 and 450 nm.

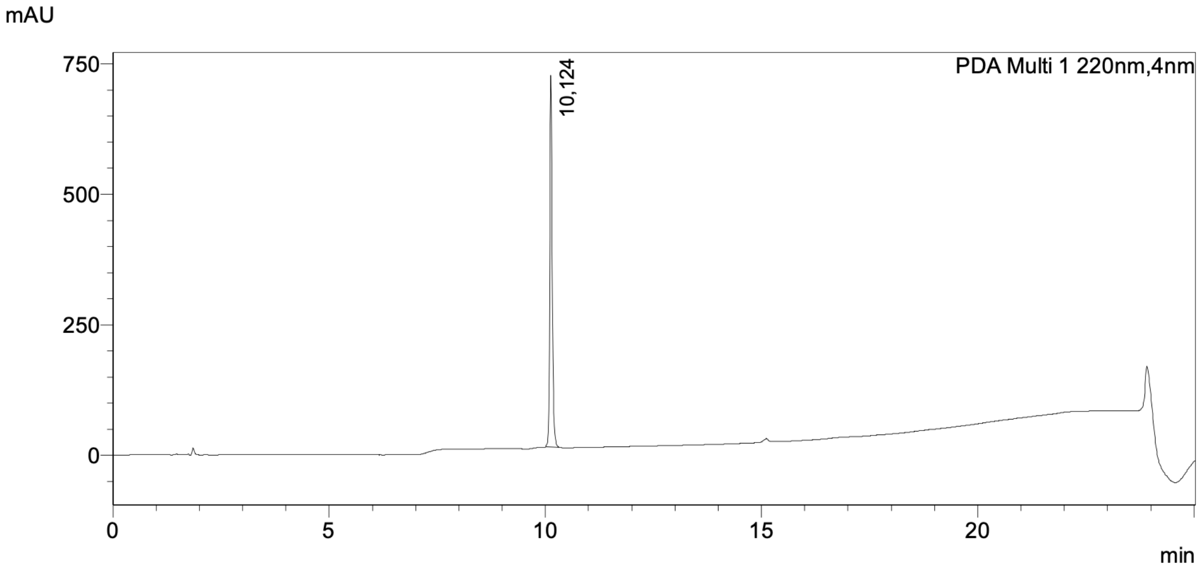

Figure S24. Chromatogram of Tat-Myb$^{397-415}$ (t_R = 10.1 min).

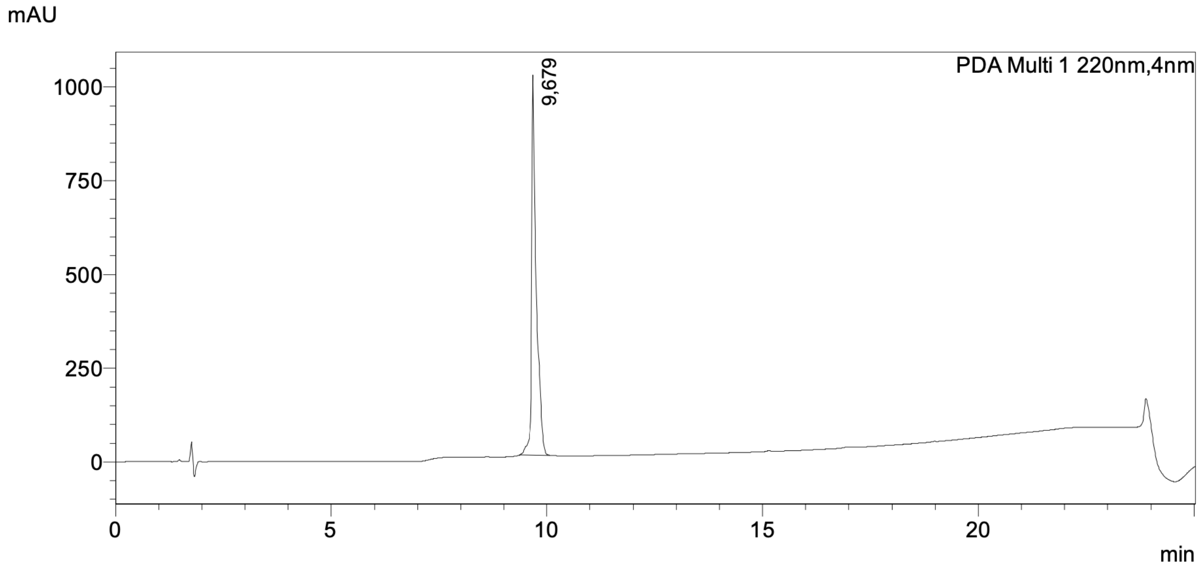

Figure S25. Chromatogram of YG-Tat-Myb$^{397-415}$ (t_R = 9.7 min).

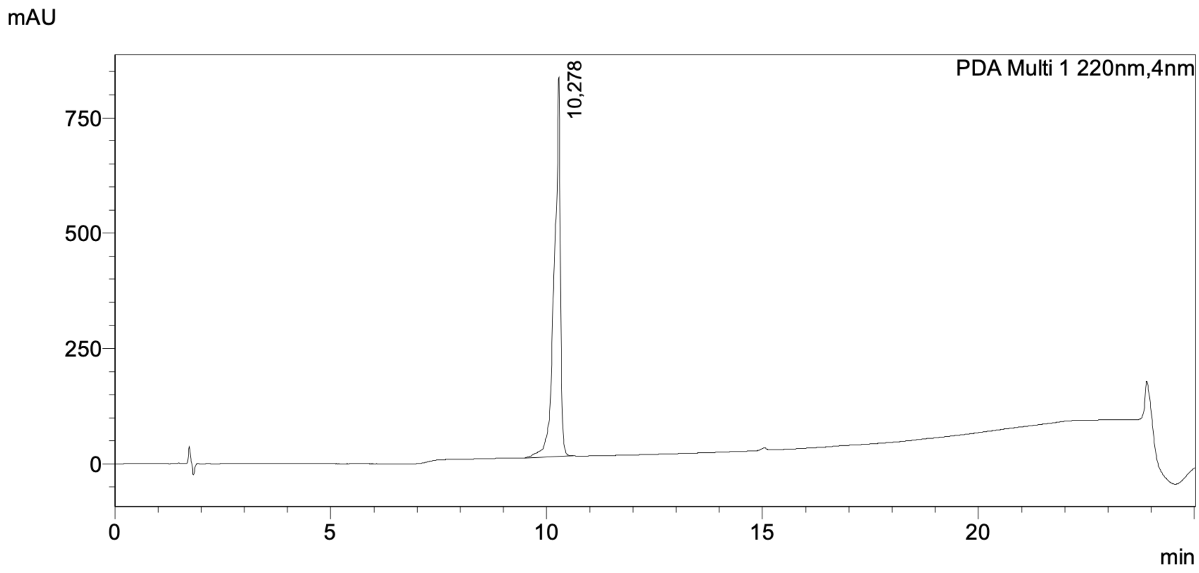

Figure S26. Chromatogram of R_6W_3-Myb$^{397-415}$ (t_R = 10.3 min).

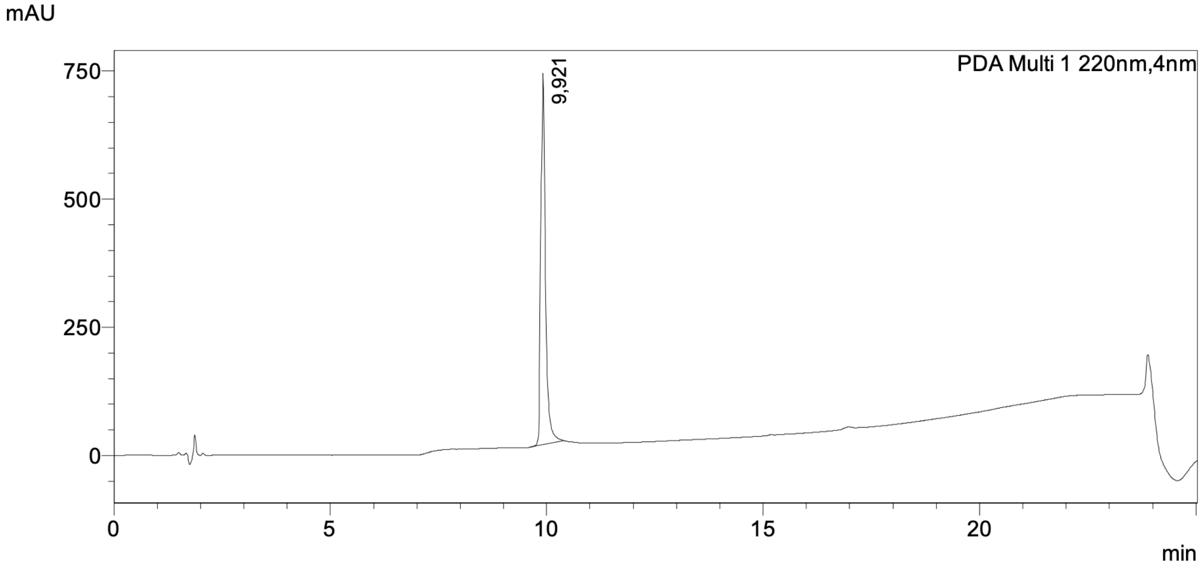

Figure S27. Chromatogram of R_7W-Myb$^{397\text{-}415}$ (t_R = 9.9 min).

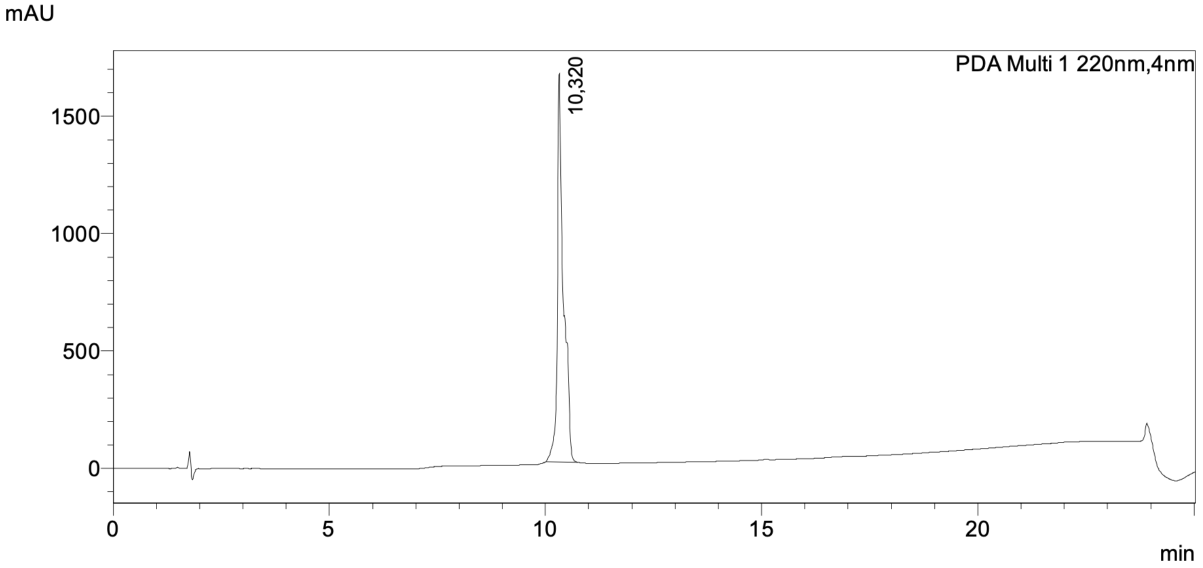

Figure S28. Chromatogram of R_7W-Myb^{A400} (t_R = 10.3 min).

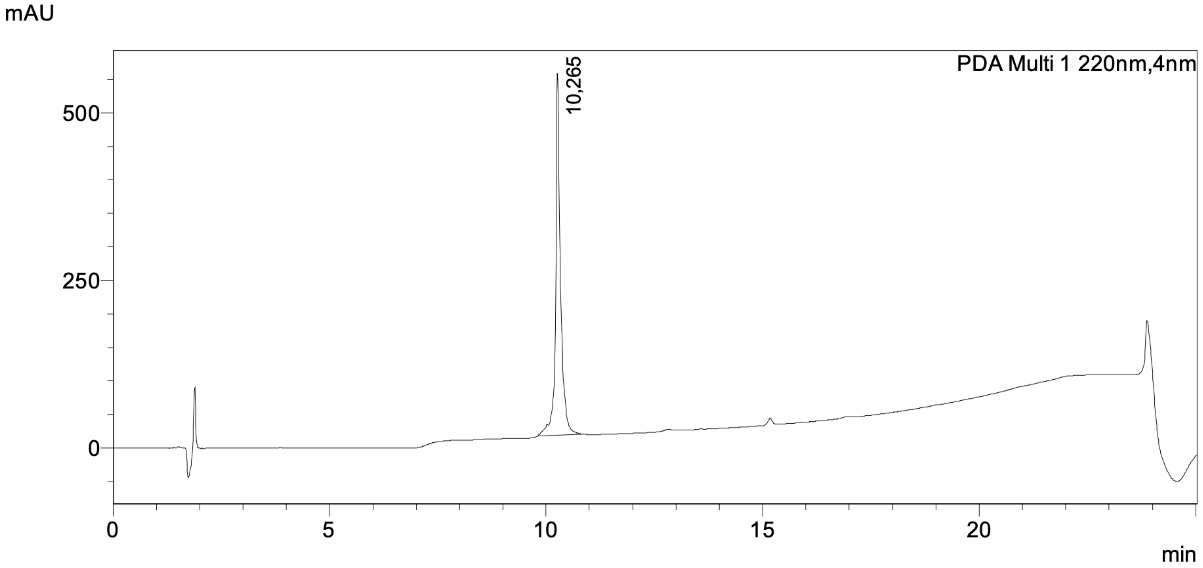

Figure S29. Chromatogram of R_7W-Myb^{A412} (t_R = 10.3 min).

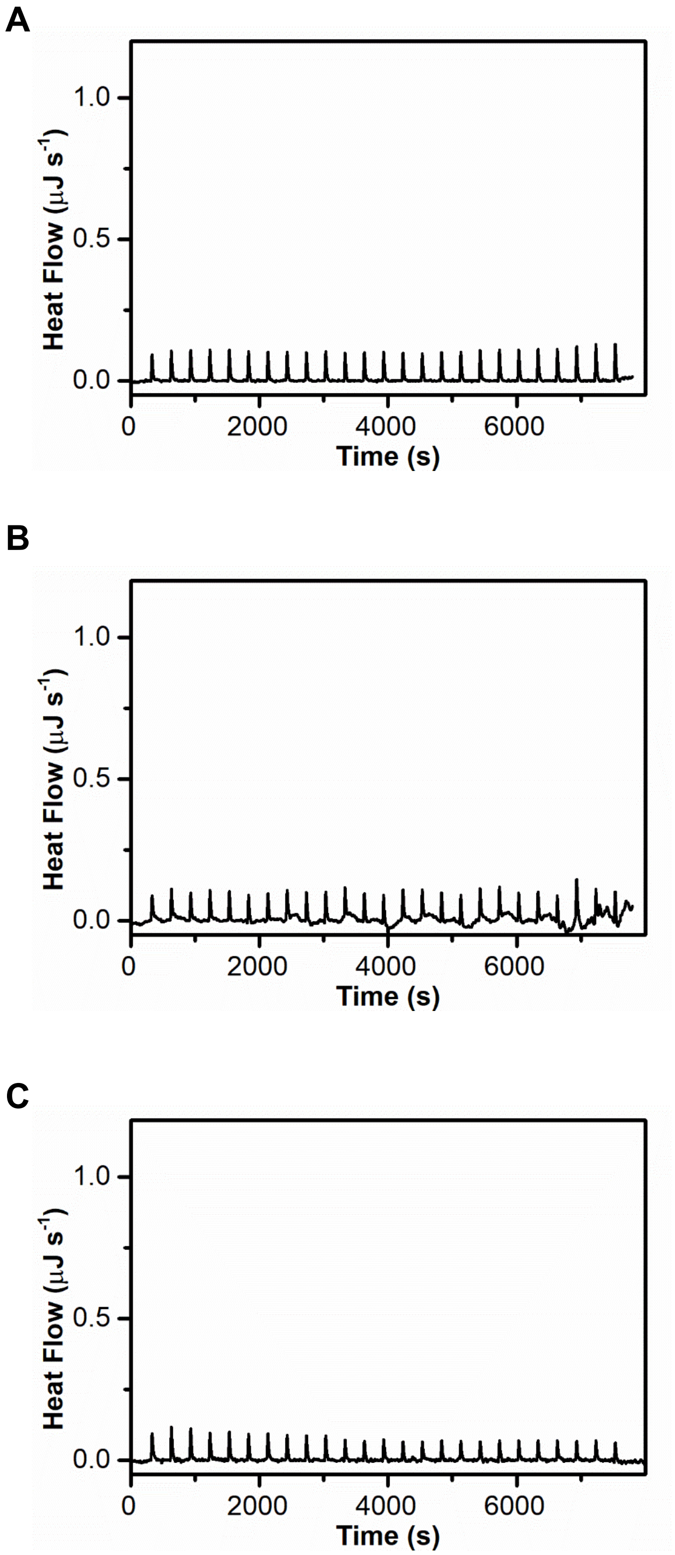

Figure S30. Raw ITC data for control experiment performed by injecting (**A**) Myb$^{397\text{-}415}$, (**B**) Myb^{A400}, and (**C**) Myb^{A412} into the buffer (5 mM KH_2PO_4/K_2HPO_4, pH 7.0, containing 20 mM KCl) at 298 K.

Table S2. List of primers used for RT-PCR.

Primer	Sequence (5'→3')
ERBB2 FWD	TTTGATGGTGACCTGGGAAT
ERBB2 REV	GAACATCTGGCTGGTTCACA
BCL2 FWD	CTGCACCTGACGCCCTTCACC
BCL2 REV	CACATGACCCCACCGAACTCAAAGA
H3 FWD	GTGAAGAAACCTCATCGTTACAGGCCTGGT
H3 REV	CTGCAAAGCACCAATAGCTGCACTCTGGAA

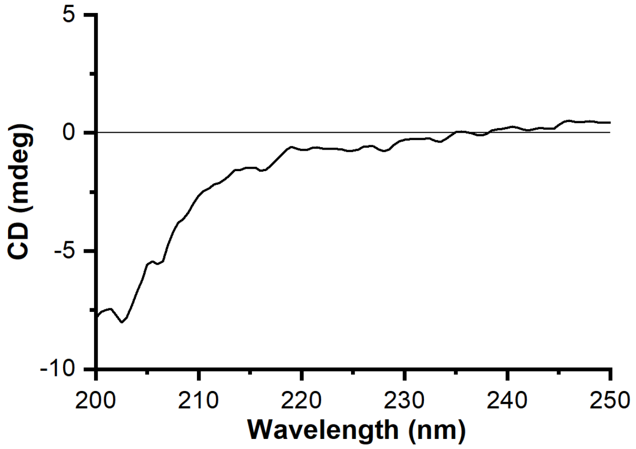

Figure S31. CD spectrum of Myb1$^{397\text{-}415}$ peptide (4 µM) recorded at 20 °C in 5 mM KH_2PO_4/K_2HPO_4 buffer containing 20 mM KCl (pH 7.0).

Parallel G4s

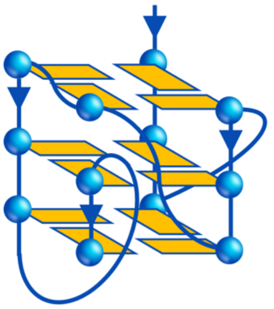

c-KIT1

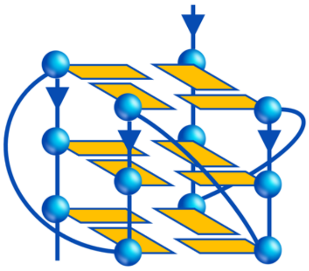

c-KIT2
c-MYC
HT-FANA

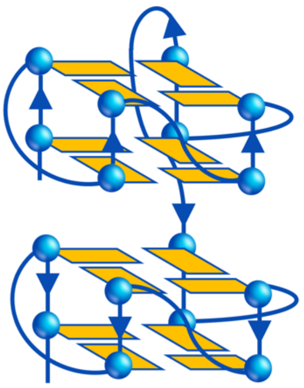

HER2

Antiparallel G4s

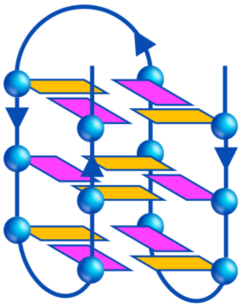

HRAS1

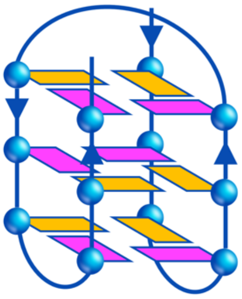

LWDLN1

Hybrid G4s

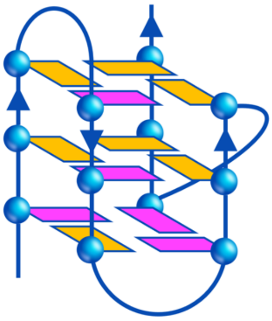

BCL-2
HT2

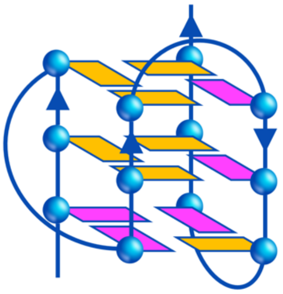

HT1

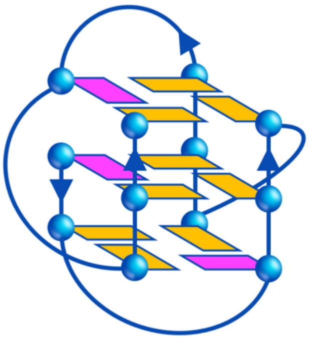

VEGFR-17T

Figure S32. Schematic representations of the different folding topologies of the investigated G4 structures. The *anti* and *syn* guanines are coloured in orange and magenta, respectively. The arrows indicate the direction of the DNA strands from the 5′ to 3′ end.

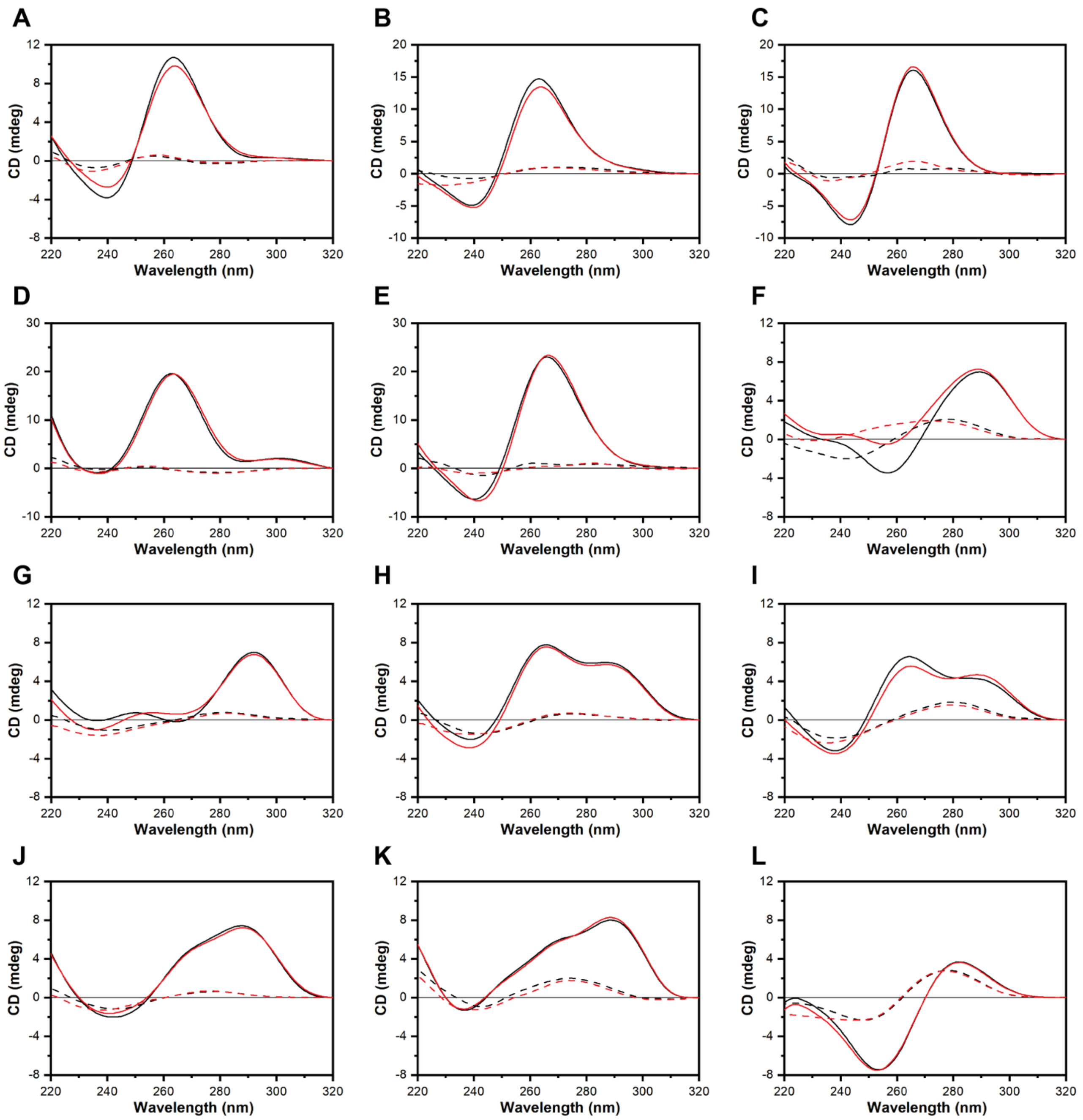

Figure S33. CD spectra of (**A**) *c-KIT1*, (**B**) *c-KIT2*, (**C**) *c-MYC*, (**D**) *HER2*, (**E**) *HT-FANA*, (**F**) *HRAS1*, (**G**) *LWDLN1*, (**H**) *BCL-2*, (**I**) *VEGFR-17T*, (**J**) *HT1*, and (**K**) *HT2* G4s, together with (**L**) ds_{12} duplex, in the absence and presence of 1 mol equiv of Myb$^{397\text{-}415}$ peptide (black and red lines, respectively), recorded at 20 and 100 °C (solid and dashed lines, respectively).

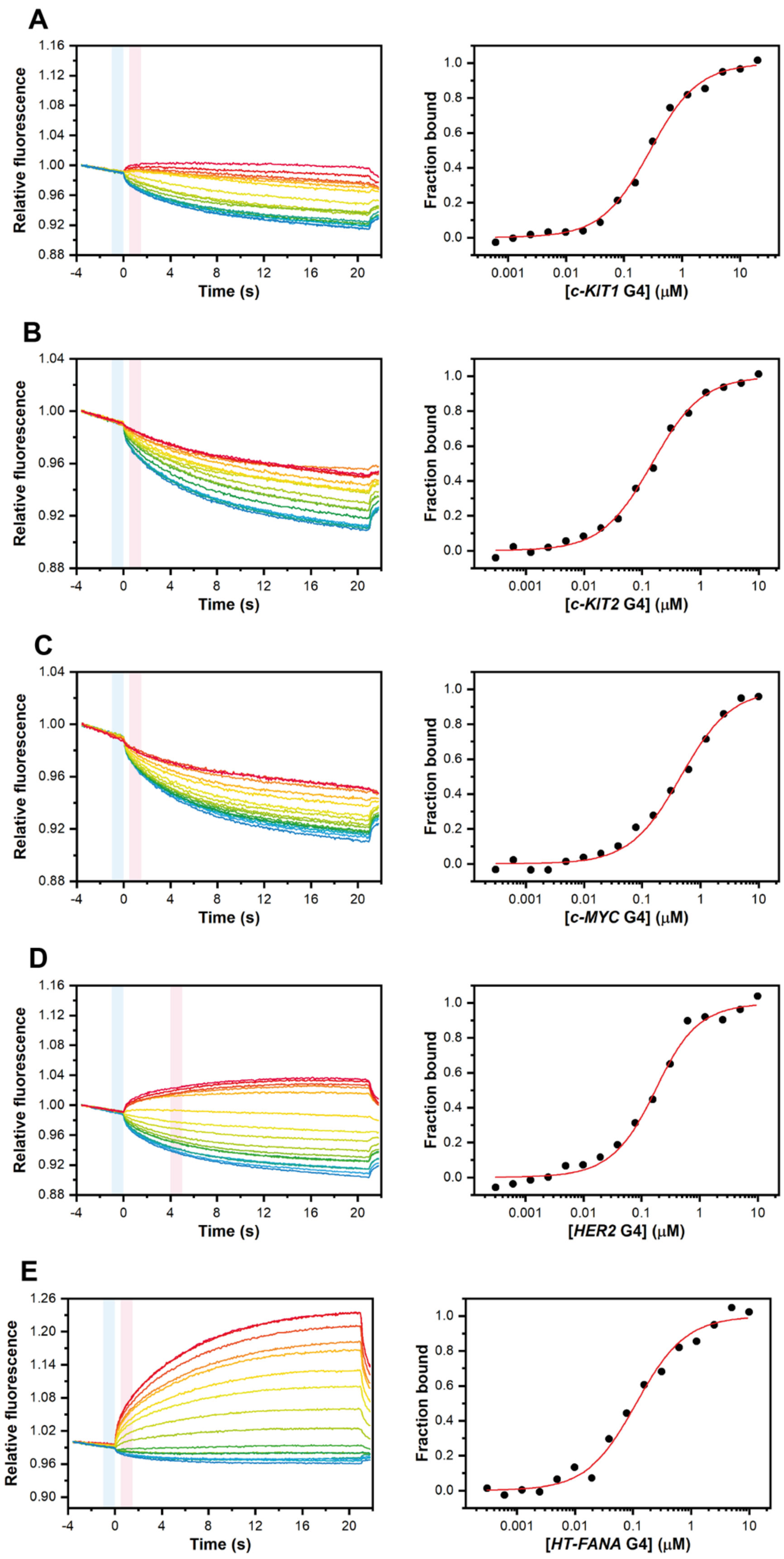
A
Relative fluorescence
Time (s)
Fraction bound
[c-KIT1 G4] (μM)
B
Relative fluorescence
Time (s)
Fraction bound
[c-KIT2 G4] (μM)
C
Relative fluorescence
Time (s)
Fraction bound
[c-MYC G4] (μM)
D
Relative fluorescence
Time (s)
Fraction bound
[HER2 G4] (μM)
E
Relative fluorescence
Time (s)
Fraction bound
[HT-FANA G4] (μM)

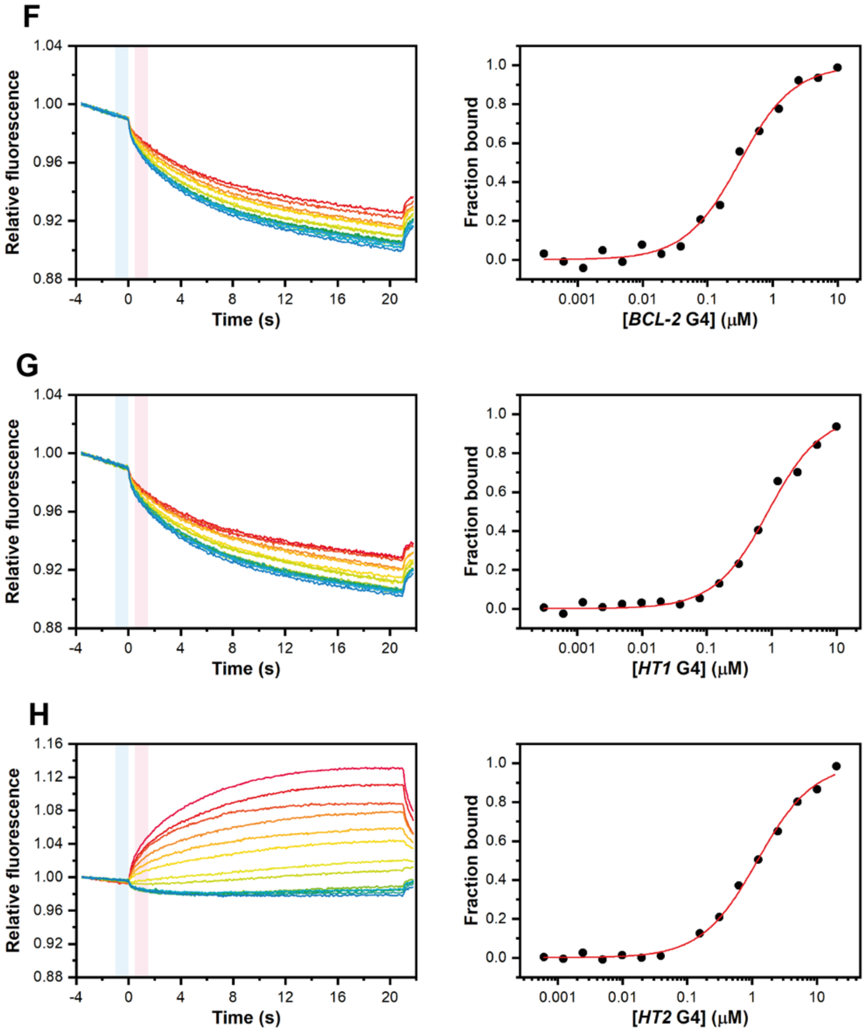

Figure S34. MST experiments for the interaction of (**A**) *c-KIT1*, (**B**) *c-KIT2*, (**C**) *c-MYC,* (**D**) *HER2*, (**E**) *HT-FANA*, (**F**) *BCL-2*, (**G**) *HT1*, and (**H**) *HT2* G4s with the fluorescein isothiocyanate-labeled peptide Myb$^{397\text{-}415}$ (FITC-Myb$^{397\text{-}415}$). (Left) Time traces recorded by adding increasing concentrations of G4s to the labeled peptide and (right) the corresponding binding curves.

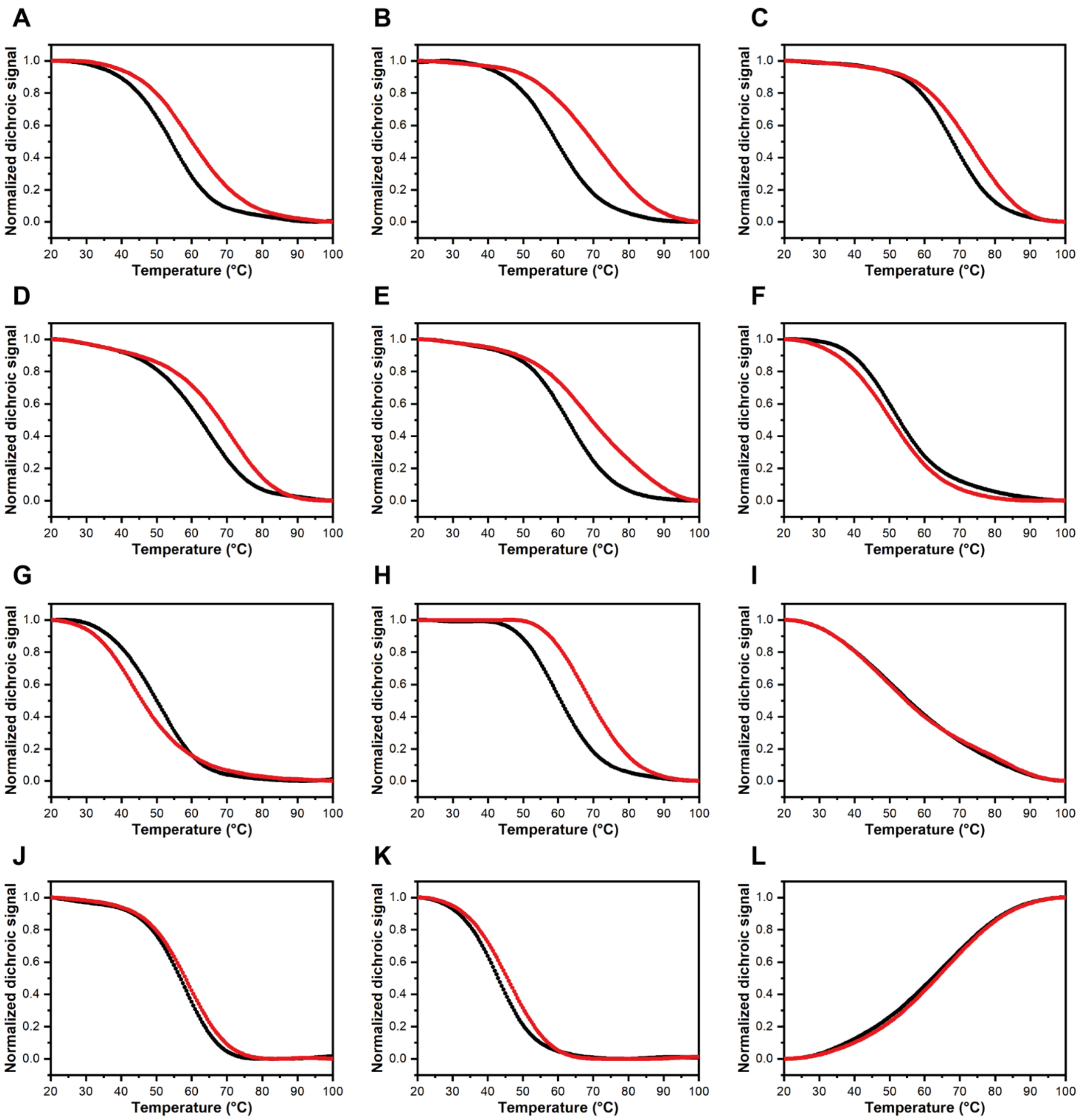

Figure S35. Normalized CD melting curves of (**A**) *c-KIT1*, (**B**) *c-KIT2*, (**C**) *c-MYC*, (**D**) *HER2*, (**E**) *HT-FANA*, (**F**) *HRAS1*, (**G**) *LWDLN1*, (**H**) *BCL-2*, (**I**) *VEGFR-17T*, (**J**) *HT1*, and (**K**) *HT2* G4s, together with (**L**) ds_{12} duplex, in the absence and presence of 1 mol equiv of Myb$^{397\text{-}415}$ (black and red lines, respectively).

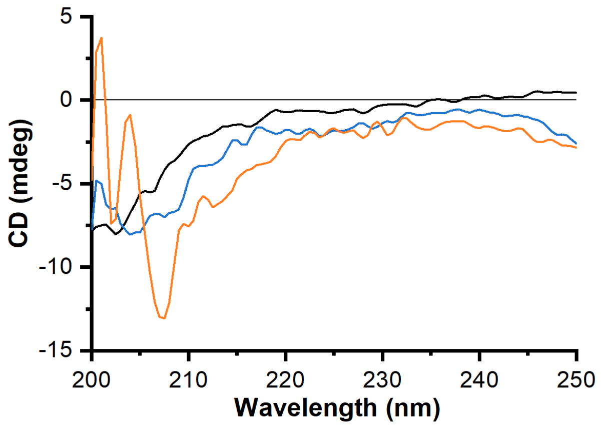

Estimated secondary structure content (%)

	Helix	Antiparallel	Parallel	Turn	Others
$Myb^{397\text{-}415}$	0.0	23.2	0.0	19.2	57.6
$Myb^{397\text{-}415}$ + *c-KIT2*	10.0	22.4	0.0	19.1	48.6
$Myb^{397\text{-}415}$ + *HER2*	22.7	20.5	0.0	13.8	43.1

Figure S36. (Top) CD spectra of $Myb^{397\text{-}415}$ (4 μM) recorded at 20 °C in 5 mM KH_2PO_4/K_2HPO_4 buffer (pH 7.0) containing 20 mM KCl in the absence (black line) and presence of *c-KIT2* and *HER2* G4s (2 μM, blue and red line, respectively). Subtraction of the G4 signal from the spectrum of each G4/peptide complex was carried out to enable the detection of conformational changes in the peptide upon G4-binding. (Bottom) Secondary structure content estimation for $Myb^{397\text{-}415}$ using BeStSel.

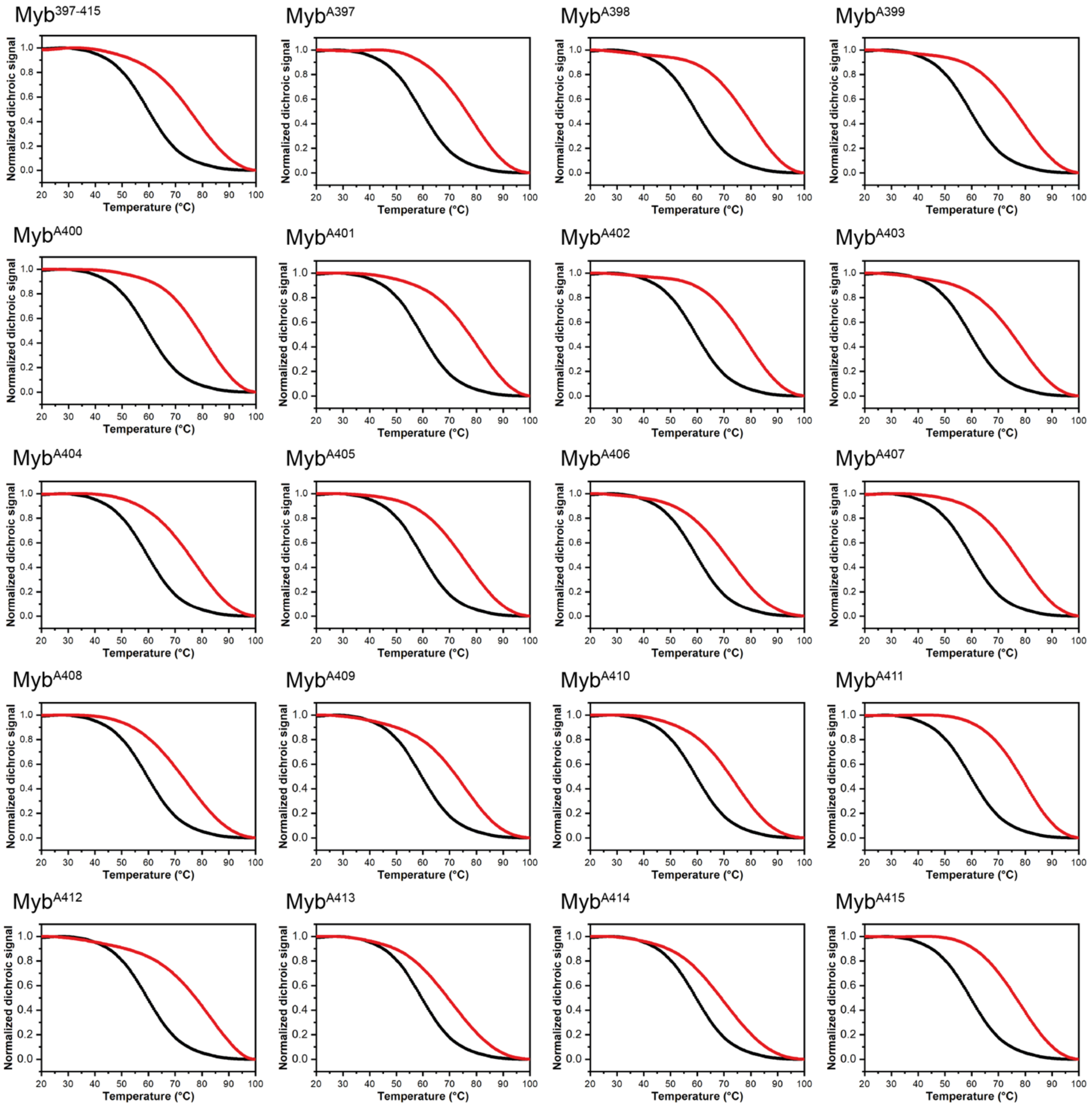

Figure S37. Normalized CD melting curves for *c-KIT2* G4 in the absence and presence (black and red lines, respectively) of 2 mol equiv of the indicated peptides.

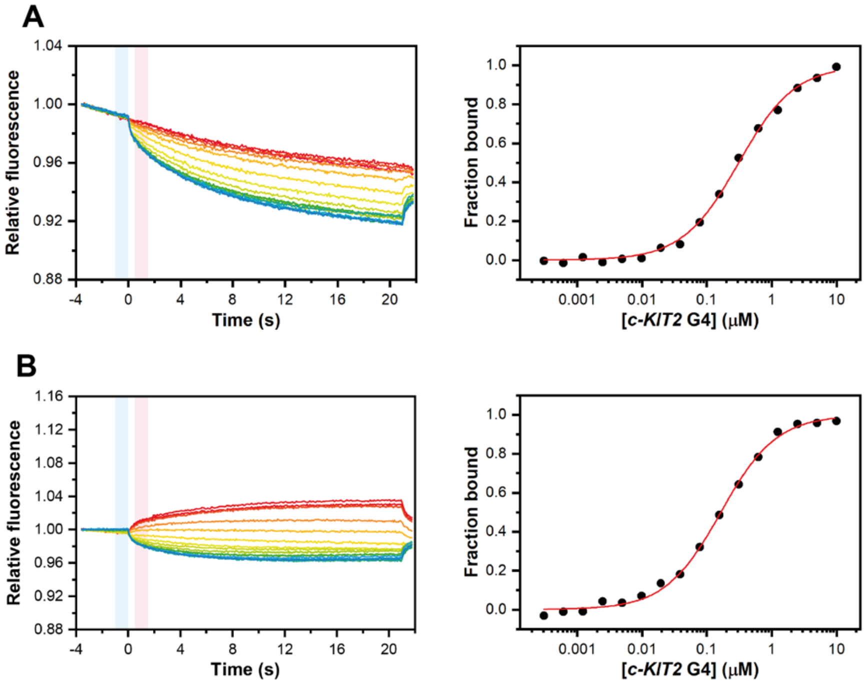

Figure S38. MST experiments for the interaction of *c-KIT2* with (**A**) FITC-Myb^{A400}, and (**B**) FITC-Myb^{A412}. (Left) Time traces recorded by adding increasing DNA concentrations to the labelled peptides and (right) the corresponding binding curves.

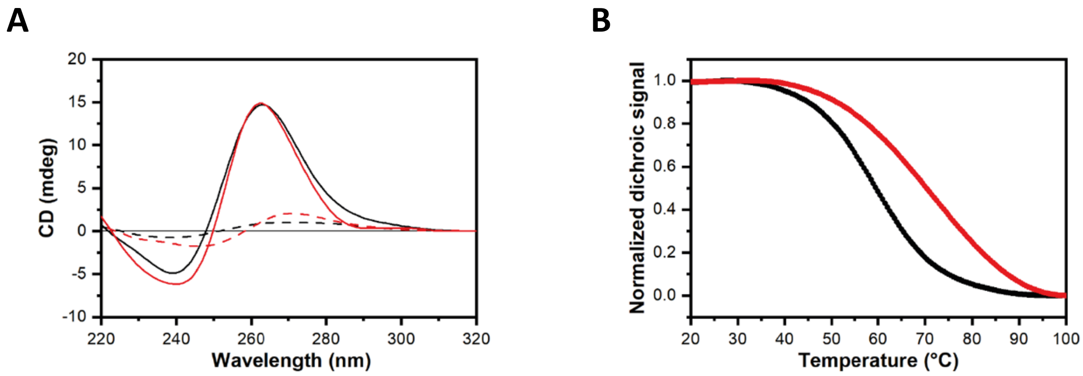

Figure S39. (**A**) CD spectra of *c-KIT2* G4 in the absence and presence of R_7W-Myb$^{397\text{-}415}$ (black and red lines, respectively) recorded at 20 and 100 °C (solid and dashed lines, respectively). (**B**) Normalized CD melting curves of *c-KIT2* G4 in the absence and presence of 1 mol equiv of R_7W-Myb$^{397\text{-}415}$ (black and red dotted lines, respectively).

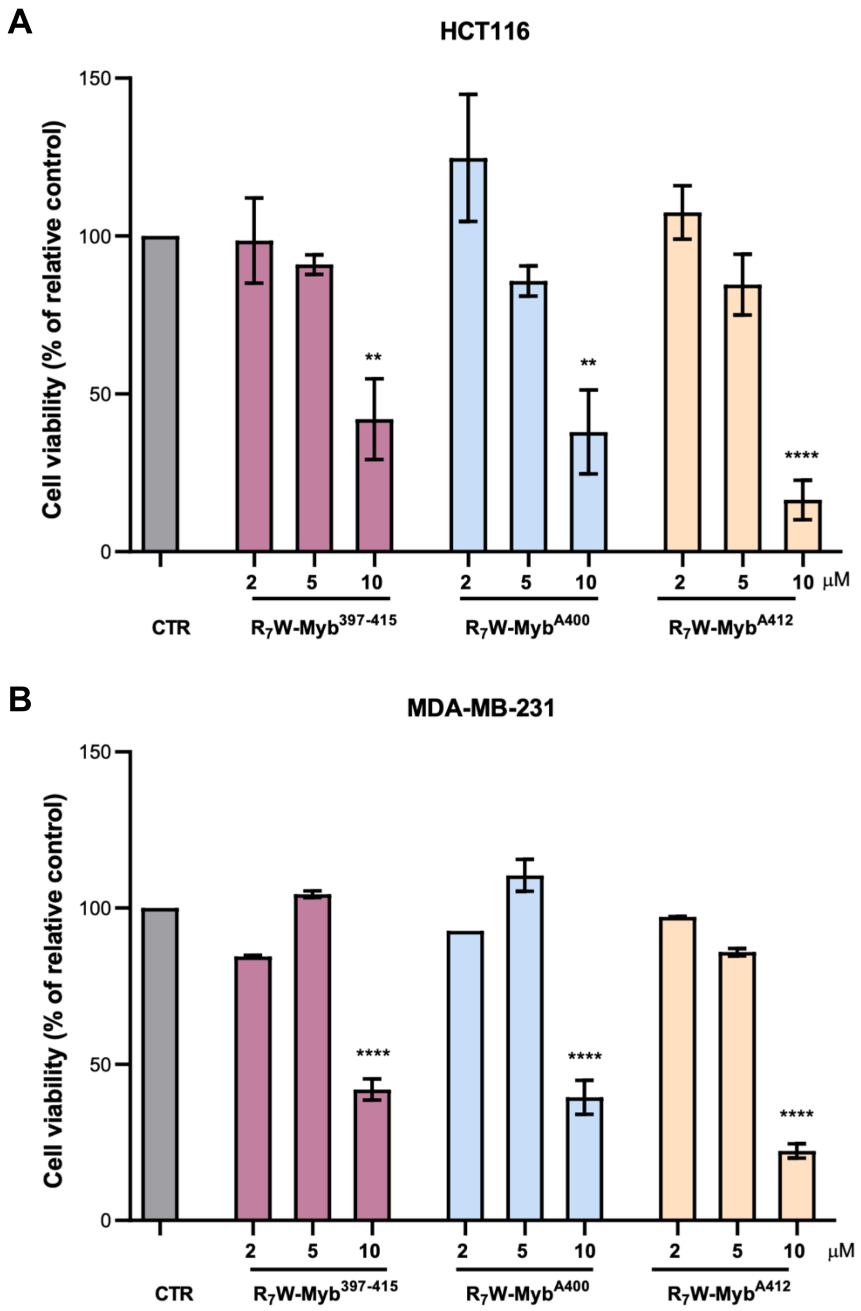

Figure S40. Antitumoral activity of the peptides evaluated in telomerase-positive BRCA proficient cell lines. (**A**) Colorectal cancer cells, HCT116, and (**B**) triple negative breast cancer cells, MDA-MB-231, were treated for 72 h with the indicated concentration of the R_7W-conjugated peptides (R_7W-Myb$^{397\text{-}415}$, R_7W-Myb^{A400}, and R_7W-Myb^{A412}) and cell viability was determined by crystal violet assay. Results are expressed as the percentage of viable cell in treated samples over their untreated control counterpart (CTR). Histograms show the mean values ±SD of three independent experiments performed in triplicate; $^{**}p<0.01$, $^{****}p<0.0001$.
